# First In Silico Study of Two *Echinococcus granulosus* Glyceraldehyde-3-Phosphate Dehydrogenase Isoenzymes Recognized by Liver Cystic Echinococcosis Human Sera

**DOI:** 10.3390/ijms262110622

**Published:** 2025-10-31

**Authors:** Facundo Ariel Agüero, Andrea Maglioco, María Pía Valacco, Alejandra Yaqueline Juárez Valdez, Emilio Roldán, Margot Paulino, Alicia Graciela Fuchs

**Affiliations:** 1 Centro de Altos Estudios en Ciencias Humanas y de la Salud, Universidad Abierta Interamericana, Buenos Aires C1270AAH, Argentina; facundo.aguero@uai.edu.ar (F.A.A.); andrea.maglioco@uai.edu.ar (A.M.); emiliojaroldan@yahoo.com.ar (E.R.); 2 Consejo Nacional de Investigaciones Científicas y Técnicas, Buenos Aires C1270AAH, Argentina; pvalacco@qb.fcen.uba.ar (M.P.V.); al.juva13@gmail.com (A.Y.J.V.); 3 Centro de Estudios Químicos y Biológicos por Espectrometría de Masa, Facultad de Ciencias Exactas y Naturales, Universidad de Buenos Aires, Buenos Aires C1121ABG, Argentina; 4 Instituto Nacional de Parasitología “Dr. Mario Fatala-Chaben”, Administración Nacional de Salud “Dr. Carlos Malbrán”, Buenos Aires C1121ABG, Argentina; 5 Facultad de Química, Departamento de Experimentación y Teoría de la Materia y sus Aplicaciones, Área Bioinformática, Universidad de la República, Montevideo CP 12900, Uruguay

**Keywords:** *Echinococcus granulosus*, glyceraldehyde 3 phosphate dehydrogenase, in silico analysis, biomarker, molecular dynamics simulation

## Abstract

Cystic echinococcosis (CE) is an endemic zoonotic disease caused by *Echinococcus granulosus*, which forms cysts in ungulates’ intermediate hosts. Humans are accidental hosts, and CE affects more than one million people worldwide. Imaging remains the diagnostic gold standard, outperforming serological methods. This study presents an in silico analysis of two glyceraldehyde-3-phosphate dehydrogenase (GAPDH) isoenzymes from *E. granulosus* (EgGAPDH), isolated from a parasite cell line (EGPE). EgGAPDHs were recognized by sera from CE patients, identified through LC-MS/MS and PCR of metacestodes from cattle liver. One isoenzyme is intracellular (IC) (UniProt: W6UJ19), and the other is extracellular (EC) (UniProt: W6V1T8). GAPDH is involved in host–parasite interactions and metabolic processes. We characterized the physicochemical properties; linear epitopes (LEPs); and amino acid domains of EgGAPDH, its hosts, and other parasites. W6UJ19 emerged as the most promising isoenzyme as a marker of infection. Molecular dynamics simulations of isoenzymes, performed in the presence or absence of two bisphosphonates (BPs), revealed how drug binding alters conformational epitopes (CEPs) and suggested that W6UJ19 is more responsive to BP modulation. Binding affinity analysis using the MMPBSA method revealed that etidronate (EHDP) binds EgGAPDH with greater affinity than phosphate (Pi) and alendronate (AL), in the following order: EHDP > Pi > AL.

## 1. Introduction

Cystic echinococcosis (CE) is a zoonotic endemic disease produced by the *Echinococcus granulosus* s.s (*E. granulosus*) in the intermediate host. *E. granulosus* s.l. belongs to the class *Cestoda*, and based on the last consensus, it is classified into four genotype clusters: *E. granulosus sensu stricto* (G1–G3), *E. equinus* (G4), *E. ortleppi* (G5), and *E. canadensis* (G6–8/10) [1]. *E. granulosus* s.s (G1/3) is prevalent in both human and livestock populations in Argentina [2]. The World Health Organization (WHO) classifies CE as a neglected disease. In 2020, it was estimated that one million people worldwide were infected, with an incidence of 50 cases per 100,000 inhabitants in hyperendemic areas. However, prevalence rates reached 5–10% in certain regions of Argentina, Peru, Central Africa, and China [3].

The life cycle of the parasite involves an intermediate host, specifically ungulate animals, and accidental hosts such as humans and cats [4]. In the intermediate host, the parasite develops cystic echinococcosis (CE) characterized by the development of a larval stage unilocular cyst, most commonly located in the liver (65–75%) and lung (23–30%) [5] and, less frequently, in the heart, bone, and pelvic organs, and other tissues. The intermediate host is infected by ingesting the oncosphere present on dust, vegetables, dog jaws, or fur. The definitive host, a member of the *Canidae* family, becomes infected by ingesting raw, infected viscera from the intermediate host. Within the intestinal lumen, the embryos develop into hermaphroditic worms that release oncospheres, which are excreted in feces after approximately 5 months of growth and maturation.

Parasitic cysts developed in the intermediate host are composed of two membranes. The outer membrane is acellular, in direct contact with the host’s tissues, and is composed primarily of glycoproteins and mucins. The inner membrane is a germinal, proliferative layer, where parasite embryos, or protoscoleces (pe), are formed through asexual sprouting. These pe are released into the cyst’s internal cavity, which is filled with hydatid fluid (HF) containing salts, proteins, and cells. The presence of the cyst induces a chronic inflammatory response in host tissue, leading to the formation of a poorly vascularized external membrane. In humans, cysts can grow up to five centimeters per year and may disseminate if ruptured due to trauma or other complications.

Cyst evolution in humans is monitored and diagnosed by abdominal ultrasonography, showing characteristic images classified according to the Gharbi classification [6], in active (I, II, and III) and inactive (IV and V). Serology is used to support diagnosis, but it is not a reliable tool for CE diagnosis or monitoring patient follow-up due to the occurrence of false-positive and false-negative results, and cross-reactions [7]. *E. granulosus* releases factors within the host that interfere with the immune response [8]. The well-characterized antigens used in CE diagnosis are AgB and Ag5. Serological methods, such as ELISA and Western blot, are performed using a homogenate of ex vivo larval tissues or HF collected from infected ungulate animals at slaughterhouses. However, the use of recombinant *E. granulosus* major antigen, rAgB, has not significantly improved the diagnostic performance of serological tests compared to native antigens [9,10].

Our group is working on the identification of new antigens from *E. granulosus* s.l. The antigenic profile of the EGPE cell line, derived from bovine *E. granulosus* G1 pe [11], demonstrated greater sensitivity in detecting serum reactivity from patients compared to HF obtained from sheep infected with *E. granulosus* G1 [12]. EGPE cells were grown in liquid medium and biphasic agarose medium, forming cystic colonies [11]. This cellular model enables the study of proteins primarily in intracellular (IC) and extracellular (EC) compartments. Using immune-identification, by CE patient sera, and LC-MS/MS methodologies, we identified four histones eluted by immune affinity from different subcellular localizations, IC and EC compartments [13].

Glyceraldehyde 3-phosphate dehydrogenase (GAPDH) is a homotetrameric enzyme of the glycolytic pathway composed of four subunits of 36 kDa. Each subunit contains an active center with the coenzyme NAD^+^ and the substrate-binding regions responsible for reducing NAD^+^ to NADH, necessary for the function of the electron transport chain and the conversion of the glyceraldehyde-3-phosphate (G3P) into 1,3-bisphosphoglycerate, which is later metabolized in the glycolytic pathway to produce ATP. GAPDH is known as a “housekeeping” gene and is localized in the cytoplasm of all animals’ somatic cells at high concentrations, accounting for approximately 5 to 15% of total soluble protein [14]. In cestodes, this enzyme has been colocalized with tegmental calcareous corpuscles, as observed in *Taenia solium* metacestodes [15]. Additionally, other roles have been attributed to GAPDH, such as vesicle and exosome biogenesis [16,17] and the binding to lactoferrin, ferritin [18], mucins [19], and fibronectin [20]. GAPDH is sensitive to free oxygen radicals, which trigger its dissociation and its later migration to the nucleus, contributing to cell death and apoptosis [21]. In *Trichomonas vaginalis*, iron availability has been shown to regulate both the synthesis and surface localization of the enzyme [22]. Moreover, multiple GAPDH isoenzymes and isoforms have also been described in various species with different organelle associations, subcellular localization, and different roles. The impact of GAPDH on parasite–host relations is probably contributing to the parasite adhesion and development, considering its presence in vesicles and exosomes secreted by the parasite [17].

BPs are synthetic analogs of bisphosphoric acid, in which the central oxygen atom is replaced by a carbon atom. These compounds exhibit pleiotropic mechanisms of action. Antiparasitic activity of BP has been proposed in *Apicomplexa*, primarily through the inhibition of farnesyl diphosphate synthase [23], interference with acidocalcisomes by mimicking the hydroxyapatite surface [24,25], and inhibition of mitochondrial ubiquinone [26]. In *Trypanosoma bruceii*, BPs have been shown to inhibit solenyl biphosphate synthase [27]. As a biological effect, BPs reduce the size of *Toxoplasma gondii* cysts in the central nervous system [28,29]. Furthermore, amino-BP have been reported to inhibit the synthesis of GAPDH in cancer cell lines [30]. The antiparasitic effects of BPs depend on both the specific compound used and the biological characteristics of the parasite.

Our research group has studied the effects of five BPs, commonly used in humans and veterinary medicine, on the EGPE cell line. We observed the inhibition of cell growth and cystic colony development upon treatment with ibandronate, etidronate (EHDP), and olpadronate. These effects were accompanied by decreases in intracellular ATP and free calcium levels [31,32]. The chemical structure of BPs determines their molecular targets. Non-amino BP, which constitute the first compound generation (e.g., EHDP), act by forming insoluble complexes with ATP. In contrast, amino-BP, such as AL, inhibit the mevalonate pathways, affecting growth factor signaling and cellular metabolism [33]. The pleiotropic actions of BPs are attributed to their chemical simplicity.

In this work, we studied two out of five GAPDH isoenzymes described in *E. granulosus* recognized by CE patient sera and identified by LC-MS/MS methodology. One was found in the IC and the other in the EC compartment. To date, no bibliographic references have been found that provide a detailed molecular characterization of EgGAPDH beyond its genetic sequences. However, studies have reported inhibitory effects of praziquantel and albendazole on capsular GAPDH in *E. granulosus* metacestodes [34]. Additionally, growth inhibition of metacestodes by antibodies against recombinant *Echinococcus multilocularis* GAPDH [35] highlights the relevance of studying this enzyme in *Echinococcus* spp.

GAPDH is one of the most representative constitutive cellular proteins, known for its multiple biological functions. In this study, two isoenzymes of EgGAPDH were investigated, one predominantly IC and the other EC. Both isoenzymes were recognized in sera from CE patients and identified by LC-MS/MS. The corresponding genes were amplified by PCR from ex vivo metacestodes of *E. granulosus* s.s. (G1 genotype). The physicochemical properties and amino acid sequence differences in identified EgGAPDH isoenzymes were analyzed and compared with GAPDH sequences from other described *E. granulosus* GAPDH, other parasites, and host organisms.

In this work, we characterized the EgGAPDH for the first time. In silico tridimensional models of both EgGAPDH isoenzymes were constructed, and LEP and CEP were predicted and mapped onto the modeled structures. Previous in vitro studies indicated that EHDP inhibited parasite cell growth, while AL did not. Then, EgGAPDH models were subjected to molecular dynamics simulations to assess whether EgGAPDH is a target of BP. After assessing their molecular stability, molecular models were used to study interactions with two BPs commonly used in human and veterinary medicine: EHDP and AL. The molecular interactions between BP and EgGAPDH may modulate the molecular dynamics of the enzyme’s quaternary structure in complex with its substrate and cofactors and alter the exposure of epitopes on the protein surface.

These results contribute to a better understanding of the structure, physicochemical properties, and immunological characteristics of EgGAPDH isoenzymes. Furthermore, this study provides insights into potential differences in the interaction of different GAPDH isoenzymes with BP, which may explain the variable efficacy of BP in inhibiting parasite cell growth in vitro and support their potential use in antiparasitic therapy.

## 2. Results

### 2.1. Characterization of EgGAPDH

#### 2.1.1. Genomic Sequences Identification

**Figure 1:**
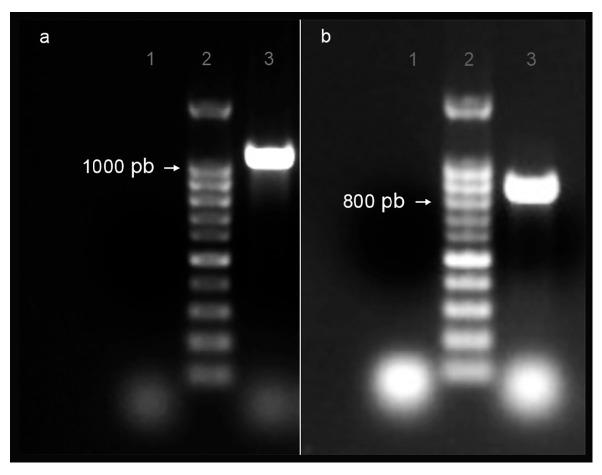
PCR products of EgGAPDH isoenzyme DNA were extracted from ex vivo liver cattle metacestode *E. granulosus* G1/s.s. (**a**) Amplification of isoenzyme W6UJ19; (**b**) amplification of isoenzyme W6V1T8. Lane 1: negative control; lane 2: Base pair marker (pb) and lane 3: PCR product.

EGPE cell proteins and supernatant proteins from EGPE colonies were recognized by antibodies from CE human sera but not by sera from patients with fasciolasis or cysticercosis. Two GAPDH isoenzymes were identified by LC-MS/MS (Project accession: PXD069559) (Table S1). Previously these enzymes were identified in ex vivo metacestodes (PXD067276). One enzyme was present in the cell homogenate (IC; UniProt W6UJ19) and another in the colony supernatant (EC; UniProt W6V1T8). DNA sequences of both GADPDH isoenzymes were obtained from *E. granulosus* ss/G1 metacestode isolated from cow liver. The nucleotide identity between the two isoenzymes was 74.11%. The PCR results are shown in Figure 1a,b.

The obtained product sequences were analyzed using BLAST (version 2.17.0) against published *E. granulosus* sequences. For isoenzyme W6UJ19, the forward sequence showed 86.74% identity with 99% coverage relative to the NCBI Reference Sequence XM_024492989.1. The reverse sequence displayed 93.53% identity with 78% coverage. In the case of isoenzyme W6V1T8, the forward sequence exhibited 81.31% identity and 72% coverage compared to the NCBI Reference Sequence XM_024494574.1. The reverse sequence showed 98.54% identity with 83% coverage (Figure S1a–d).

#### 2.1.2. Amino Acid Sequence’s Identity

Amino acid sequence identity with other *E. granulosus* GAPDH isoenzymes, none of which were recognized by human serum in reactivity assays, was evaluated. Isoenzymes W6UPZ5 and A0A068WMZ6 (UniProt) showed 26.42% and 28.63% identity, respectively, with W6UJ19 and 25.87% and 27.53% identity with W6V1T8. In contrast, isoenzyme A0A058WSX5 showed 99.70% identity with W6UJ19 and 73.13% identity with W6V1T8. Isoenzymes A0A068WKQ2 (UniProt) and A0A096ZQK3 (UniProt) exhibited 71.64% and 73.12% identity with the IC isoenzyme (W6UJ19), and 96.13% and 99.79% identity with the EC isoenzyme (W6V1T8).

The amino acid sequences of the EgGAPDH isoenzymes W6UJ19 and W6V1T8 were compared with GAPDH sequences from other species. W6UJ19 showed the following sequence identities: *E. multilocularis* 71.73%, *T. solium* 75.6%, *Fasciola hepatica* 73.94%, *L. mexicana* 53.43%, *H. sapiens* (UniProt P04406) 53.43%, *Bos taurus* 70.57% and *Ovis aries* 70.81%. In contrast, W6V1T8 shared higher sequence identities with most of these species: *E. multilocularis* 97%, *T. solium* 91.96%, *F. hepatica* 81.91%, *H. sapiens* 70.78%, *B. taurus* 75.38% and *O. aries* 75.78%. The exception was *L. mexicana*, with which W6V1T8 shared a lower identity (51.80%) compared to W6UJ19.

#### 2.1.3. Physicochemical Characterization

All the GAPDH isoenzymes analyzed were predicted to be thermostable and to lack a classical signal peptide for secretion. The predictive half-lives for W6UJ19, W6V1T8, and *L. mexicana* GAPDH were similar, with estimated values of 30 h in vitro in mammalian reticulocytes, over 20 h in yeast, and more than 10 h in *Escherichia coli*, according to ProtParam analysis. Despite these similarities, notable differences were observed in the predicted physicochemical properties between the *E. granulosus* isoenzymes. W6V1T8 exhibited a higher net positive charge, greater hydrophobicity, and a higher isoelectric point compared to W6UJ19 (Table 1).

### 2.2. EgGAPDH Secondary and Tertiary Structure Stability

#### 2.2.1. Secondary Structure

**Figure 2:**
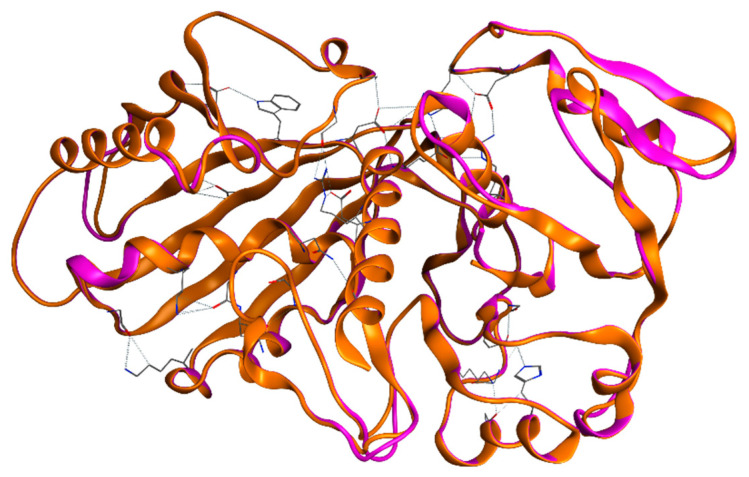
Secondary structure of EgGAPDH monomer. The image shows the superimposition of one subunit of each EgGAPDH isoenzyme: W6UJ19 (intracellular, shown in pink) and W6V1T8 (extracellular, shown in orange), as obtained using MOE.

The secondary structures of both EgGAPDH were found to be highly similar. For W6UJ19 and W6V1T8, the predicted structures were as follows: α-helix, 33.80% and 33.93%; extended strand, 23.67% and 24.70%; β-turn, 9.17% and 8.33%; and random coil, 31.36% and 33.04%, respectively (Figure 2).

#### 2.2.2. Tertiary Structure of EgGAPDH Isoenzymes Stability

The subunit of each isoenzyme was assayed for molecular structure stability. The root mean square deviation (RMSD) between the ab initio models generated with Phyre2 and the AlpHafold3 models was 1.27 Å for W6UJ19 and 1.42 Å for W6V1T8. After 100 ns of molecular dynamics simulation, the tertiary structures remained within a stable conformational range, indicating consistent structural stability throughout the simulation (Figure S2).

#### 2.2.3. Quaternary Structures and LEP of Isoenzymes

**Figure 3:**
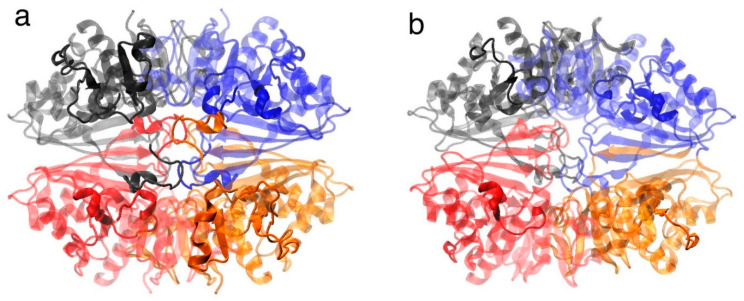
Quaternary structure and LEP mapping of EgGAPDH isoenzymes. In (**a**), the intracellular isoenzyme W6UJ19 is shown; in (**b**), the extracellular isoenzyme W6V1T8 is shown. Both quaternary structures are shown as homotetramers, with the four subunits depicted in colors. LEPs are highlighted in opaque. Each color represents one monomer.

Monomers of both isoenzymes were assembled in a quaternary structure through non-covalent bonds (Figure 3a,b).

The sequences of the predicted EgGAPDH LEPs were compared with the amino acid sequences of GAPDH from other species, including *E. multilocularis*, *T. solium*, *F. hepatica*, *L. mexicana*, *H. sapiens*, *B. taurus*, and *O. aries*. No similarities were found with the intracellular isoenzyme of GAPDH. However, the extracellular isoenzyme W6V1T8 showed similarity with *T. solium* (UniProt A8R8Q4) in the LEP region K138-T153, and with *E. multilocularis* (UniProt Q27652) in regions K138-T153 and F286-I301.

- Post-translational LEP modifications.

The complete enzyme is formed by the assembly of four identical subunits without covalent bonds. LEPs were predicted for both W6UJ19 and W6V1T8. Table 2 shows the amino acid sequences of the LEP along with their predicted post-translational modifications.

### 2.3. Isoenzymes Docking and Molecular Dynamics Simulation

#### 2.3.1. Isoenzymes Docking

**Figure 4:**
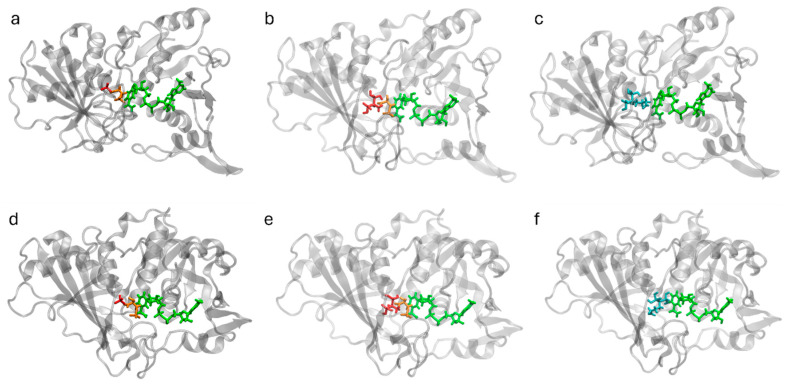
EgGAPDH and ligands docked in subunit A. Top row: GAPDH W6UJ19 (intracellular) from left to right: (**a**) protein with G3P (orange), Pi (red), and NAD^+^ (green); (**b**) G3P (orange), EHDP (red), and NAD^+^ (green); (**c**) AL (cyan) and NAD^+^ (green). Bottom row: GAPDH W6V1T8 (extracellular) from left to right: (**d**) protein with G3P (orange), Pi (red), and NAD^+^ (green); (**e**) G3P (orange), EHDP (red), and NAD^+^ (green); (**f**) AL (cyan) and NAD^+^ (green).

GAPDH is composed of four identical subunits, each containing an active site for substrate binding, G3P, and binding sites for NAD^+^ and Pi (Figure 4). These functional sites were conserved among the GAPDH proteins analyzed in this study. However, *L. mexicana* and *O. aries* showed greater variability in the conformational binding sites for NAD^+^ and Pi (Table S2).

- Isoenzymes potential energy

The potential energy of both isoenzyme control systems (quaternary structure of EgGAPDH with the substrate, Pi, and NAD^+^) through 100 ns of dynamic simulation is summarized in Table 3. Notably, the total potential energy exhibits a negative value and remains stable across all the simulations. The electrostatic partition constitutes the largest and most negative component. Conversely, the Van der Waals energy, although serving as a positive counterpart, possesses a relatively minor absolute value. Consequently, the overall energy persists in a negative state, underscoring the stability of the system. This outcome is consistent across all the simulations, reinforcing the notion that the electrostatic contribution plays a dominant role in determining the system’s energetic properties. Overall, the potential energy profiles of both isoenzymes in the control, EHDP, and AL systems are shown in Figure S3.

#### 2.3.2. Amino Acids of EgGAPDH Involved in Substrate, Pi, and NAD^+^ Binding During Dynamics Simulation

**Figure 5:**
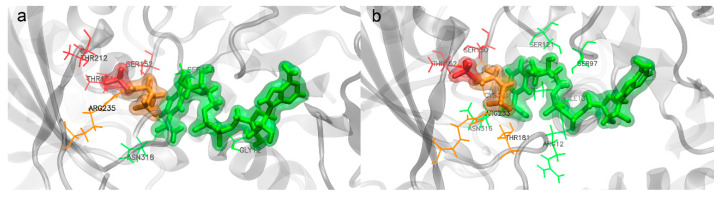
Ligand interactions of Pi (red), G3P (orange), and NAD^+^ (green) in subunit A of EgGAPDH: (**a**) IC isoenzyme W6UJ19 and (**b**) EC isoenzyme W6V1T8. Residues are highlighted in sticks, in the same color as the ligands they interact with.

Throughout the molecular dynamics simulations, each subunit of EgGAPDH showed two binding sites corresponding to the substrate, Pi, and NAD^+^. These two sites corresponded to the beginning or the end of the 100 ns simulation. While the binding sites involved similar amino acids overall, differences were observed between isoenzymes and among subunits. The amino acids involved in substrate, Pi, and NAD^+^ binding for the IC isoenzyme are shown in Figure 5a, and for the EC isoenzyme in Figure 5b. Interaction details in the 2D ligand interaction plot are shown in Figures S4 and S5.

- Substrate binding

The residue R233 or R235 was involved in substrate binding of the monomers A, B, and C in the IC and monomers A and B of the EC isoenzyme at the beginning of the simulation. At the end of the simulation, this binding was maintained in monomers A and B in the IC, and appeared in all the monomers (A, B, C, and D) of the EC isoenzyme.

- Inorganic phosphorus binding

For Pi binding, R233 was involved at the end of the simulation in monomers A, B, and C of the IC isoenzyme. In contrast, for the EC isoenzyme, R233 was involved in Pi interaction only in subunit C, with no Pi binding observed in the other monomers at the end of the simulation.

- NAD^+^ binding

NAD^+^ binding interactions presented greater variability between subunits at the initial time point than at the end of the simulation, particularly in subunit A of the IC isoenzyme. Subunit C of the EC isoenzyme displayed different NAD^+^ interacting residues between the start and the end of the simulation, whereas subunit D showed no interaction with NAD^+^ at the end of the simulation.

#### 2.3.3. Binding Free Energy (∆G) for Substrate, Pi, and NAD^+^

The binding free energy (∆G) values were calculated using the MMPBSA method based on the last 100 frames of the simulation. The binding free energy varied among the subunits of each EgGAPDH isoenzyme, highlighting that the amino acid interaction pattern may differ between subunits (Table S3). The binding of the substrate to monomer A yielded a ∆G binding: −10.6968 ± CI 95% 1.005 for W6UJ19 and −4.72 ± CI 95% 0.64 for W6V1T8. In contrast, NAD^+^ binding resulted in negative ∆G values only in monomers B (−4.0176, CI 95% ± 0.725) and D (−0.61, CI 95% ± 0.55) of W6V1T8. The calculated ∆G values for Pi were positive across all the subunits and isoenzymes.

### 2.4. Interaction of BP with Both EgGAPDH Isoenzymes

Two BPs, AL and EHDP, were docked in silico, showing stable molecular interactions with both EgGAPDH isoenzymes. These interactions did not alter the interaction potential energy, which remained consistent with the values shown in Table 3 and Figure S3. The structural stability of both enzymes in the presence of each BP is shown in Figure S6a,b through RMSD profiles.

Despite similar structural stability, differences were observed in the interactions between the two BPs and EgGAPDH. AL did not support co-binding of the substrate and Pi, whereas EHDP was able to dock with G3P at the active site, although it did not support Pi interaction.

#### 2.4.1. Docking Affinity

- EHDP increased Pi docking

After docking the EHDP in the presence of NAD^+^, Pi, and G3P, establishing an interaction zone (docking site) consisting of a sphere at 4.5 Å centered on G3P, we observed that the EHDP was positioned superimposed on the Pi, so a new situation was modeled in which the EHDP is positioned in the same place as the Pi, having displaced the Pi.

Using the “placement none” resource, which allows the static interaction situation to be measured in each case, the interaction energy of the Pi in the NAD^+^, Pi, G3P complex in the absence of the EHDP was measured (Table 4):

- AL increased NAD^+^ docking

After docking with AL in both enzymes, containing G3P, Pi, and NAD^+^, it was observed that AL was positioned in the same area as G3P and Pi. Therefore, a putative model was generated of an enzyme that is blocked by AL, having displaced itself during the interaction of all components to G3P and Pi. This does not mean that the docking has caused such displacement. This only proposes a possible model that could explain the interaction between AL and NAD^+^.

In these putative models, AL showed different influences on the binding of NAD^+^ at the site in both EgGAPDH isoenzymes (Table 5).

#### 2.4.2. BP Effect on the Isoenzyme Substrate Affinity Studied by Molecular Dynamics

A measurement of the free energy differences (∆G) of ligands and enzymes was calculated by the MMPBSA method. Table 6 shows that the addition of BP changed the subunit affinity for the substrate, NAD^+^ and Pi, accordingly with EHDP or AL presence.

- Substrate affinity.

Upon EHDP addition, substrate binding affinity became evident in both isoenzymes. Conversely, AL inhibited G3P binding at the active site, showing only weak binding in subunit B of W6UJ19 and subunit D of W6V1T8. Both GAPDH isoenzymes exhibited a variable substrate affinity, with some subunits showing weak or no binding for the substrate depending on the studied subunit (G3P) (Table 6). This instantaneous measurement makes evident the diversity among subunits with respect to their binding.

- Pi affinity

The Pi interaction may be very transient with both isoenzymes, and its binding could not be reliably detected under control conditions. However, EHDP was able to bind to the Pi site of both isoenzymes, although binding to W6V1T8 occurred with lower affinity. In contrast, AL exhibited weak binding to the Pi conformational site on both W6UJ19 and W6V1T8 (Table 6).

- NAD^+^ affinity

No NAD^+^ binding was detected in W6UJ19, either in the presence or absence of EHDP. In W6V1T8, NAD^+^ binding was observed, and upon EHDP addition, detectable NAD^+^ binding was observed in another subunit. However, AL promoted NAD^+^ binding in W6UJ19, specifically in subunits C and D, and in W6V1T8 in subunit D, although with a weaker value than in the control subunit B (Table 6).

#### 2.4.3. Amino Acids Involved in BP Interactions

In Table 7 and Table 8, all amino acids interacting with all ligands are listed.

- EHDP and AL interactions with EgGAPDH W6UJ19 and W6V1T8

**Figure 6:**
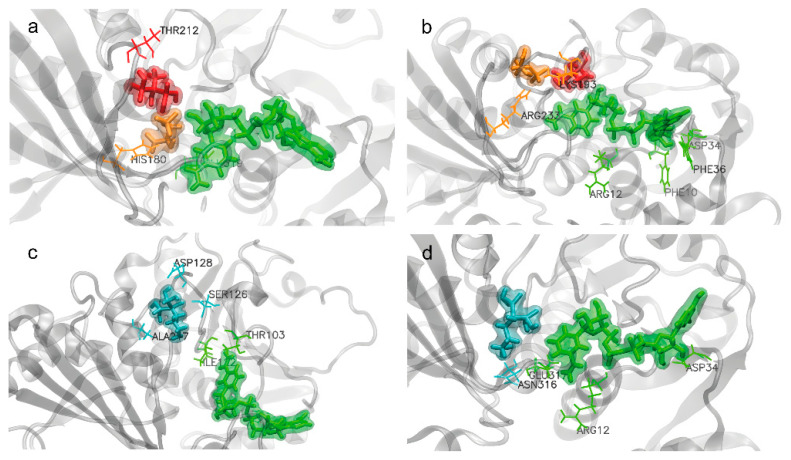
Ligand interaction at the end of simulation (100 ns). G3P (orange), EHDP (red), and NAD^+^ (green) are shown interacting with EgGAPDH W6UJ19 (**a**) and W6V1T8 (**b**). AL (cyan) and NAD^+^ (green) are shown interacting with EgGAPDH W6UJ19 (**c**) and W6V1T8 (**d**). Residues are highlighted in sticks in the same color as the ligand they interact with.

In Figure 6, the main interactions of EHDP and AL with EgGAPDH W6UJ19 and W6V1T8 are shown. Figure S7 shows a 2D ligand interaction plot between EHDP and W6UJ19. In Figure S8 a 2D ligand interaction plot between EHDP and W6V1T8 is shown. In Figures S9 and S10, the 2D ligand interaction plots between AL with W6UJ19 and W6V1T8 are shown.

### 2.5. CEP and Its Modification by the BP Interactions

#### CEPs

**Figure 7:**
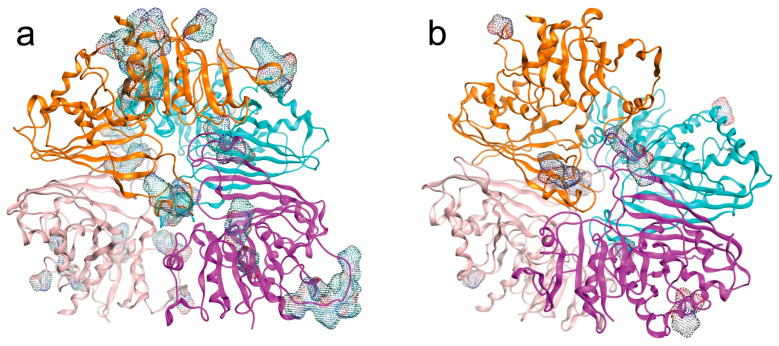
CEPs of EgGAPDH isoenzymes. Homotetramers are illustrated with CEPs mapped onto the molecular surface as dots colored according to atomic elements. Chain A is shown in orange, chain B in purple, chain C in cyan, and chain D in gray. (**a**) IC isoenzyme W6UJ19 and (**b**) EC isoenzyme W6V1T8.

Surface CEPs were identified in both isoenzymes (Figure 7a,b)

The IC isoenzyme (UniProt W6UJ19) exhibited a greater number of conformational antigenic sites than the EC isoenzyme (UniProt W6V1T8). Subunit A was the most antigenic in the intracellular isoenzyme, while subunit B was the most antigenic in the extracellular isoenzyme based on the number of amino acid residues exposed in CEP (Table 9).

Although BP established non-covalent interactions with amino acid residues, they altered the surface exposure of the CEP. The effect of BP on the number and composition of amino acids in the CEP was most evident with EHDP on W6UJ19. The interaction of BP with CEPs of both isoenzymes is summarized in Table 9.

## 3. Discussion

GAPDH is a housekeeping protein, highly represented in cells with regulated enzymatic activity whose transcription and intracellular localization are regulated by feeding or cell proliferation [36]. GAPDH is a highly conserved protein throughout evolution, functioning in the glycolysis pathway by catalyzing the conversion of G3P, NAD^+^, and Pi into 1,3-bisphosphoglycerate and NADH, thereby connecting glycolysis with the respiratory chain. The enzymatic activity depends on the integrity of its quaternary structure, sustained by NAD^+^ interaction and the absence of negative cooperativity among its monomers, as demonstrated in studies on yeast GAPDH [37]. However, several additional functions have been attributed to GAPDH in cellular biology, including roles in endocytosis [38], fusion of plasma [39] and nuclear membranes [40], and autophagy [41]. Furthermore, partially oxidized GAPDH acylphosphatase activity, which may contribute to glycolysis by coupling with oxidative phosphorylation [42]. Depending on its subcellular localization, GAPDH can perform distinct functions. Nuclear GAPDH, whose localization is cell cycle-dependent, is proposed to play a protective role against DNA damage at telomeres caused by ceramide and toxins [43].

In addition, GAPDH contributes to cellular adaptability and defense against pathogens by participating in protein biogenesis and the regulation of gene expression [44]. Extracellular GAPDH has also been implicated in host–parasite interaction; for example, during leishmaniasis infection, it has been shown to downregulate TNF-α expression in host cells [45].

Given its multiple roles and physiological functions, GAPDH has been proposed as a pharmacological target in cancer, neurological diseases, and chronic parasitic infections [34]. Despite its high evolutionary conservation, GAPDH can elicit an immune response. Recombinant *E. multilocularis* GAPDH has been tested in laboratory animals to prevent metacestode development [35].

Standardized and reliable serological markers are needed for the diagnosis, prognosis, and post-treatment follow-up of patients with CE infection. Moreover, if the antigens are related to metabolic processes, parasite viability, or growth, such markers could also aid in CE infection stratification. Our group is searching for novel antigenic proteins to serve as serological markers for the diagnosis and monitoring of CE. This is the first work presenting the in silico model of two GAPDH isoenzymes from *E. granulosus* ss/G1, recognized by sera from CE patients with liver cysts. These isoenzymes were found in different subcellular localizations, W6UJ19 (UniProt) in IC and W6V1T8 (UniProt) in EC. Physicochemical differences between the two isoenzymes were observed, particularly in their isoelectric points and net molecular charges, both of which were higher in the EC isoenzyme. Both isoenzymes were detected by PCR in ex vivo metacestode samples. The genetic identity between IC and EC isoenzymes was approximately 74%. In contrast, other EgGAPDH isoenzymes not recognized by immunosera (UniProt W6UPZ5 or A0A068WMZ6) shared only ~20% identity with the IC and EC isoenzymes.

Regarding LEP singularity, the IC isoenzyme W6UJ19 is more specific for *E. granulosus* than the EC W6V1T8. In fact, the EC EgGAPDH is likely responsible for the initial activation of the host immune system, producing immune cross-reaction among *E. granulosus*, *E. multilocularis*, and *T. solium*, while the IC isoenzyme, which is more specific, may be released as a consequence of parasite cell death. The IC isoenzyme (UniProt W6UJ19) emerged as the main candidate for differential diagnosis, showing approximately 53% sequence identity with the human GAPDH and approximately 70% identity with GAPDH sequences from *E. multilocularis*, *T. solium*, and *F. hepatica*, and 55% identity with *L. mexicana*. The IC isoenzyme (UniProt W6UJ19) is also the most promising candidate for constructing a recombinant multiepitope antigen. Notably, it does not share predicted LEP with *E. multilocularis* or *T. solium* in the regions spanning residues S123-L138 and F288-I303. Additionally, no post-translational modifications were predicted in the sequences containing these epitopes. Moreover, an antibody reactivity rate between W6V1T8 and W6UJ19 could be useful to determine infection prognosis and treatment success [46]. Due to analysis of metacestode vesicles by the LC-MS/MS method with different metabolic activities, correlation was found between W6V1T8 abundance (emPAI) and vesicle metabolic activity. Instead, W6UJ19 showed consistent relative abundance regardless of metabolic activity [46]. Enzymes from the glycolytic pathway are localized in calcareous corpuscles [15], where EgGAPDH EC is probably localized.

CEP exhibited results conceptually similar to those described for LEP. Those results, regarding LEP and CEP, showed that the IC isoenzyme could be more antigenic than the EC isoenzyme. The IC isoenzyme (UniProt W6UJ19) exhibited a greater number of conformational antigenic sites than the EC isoenzyme (UniProt W6V1T8). Moreover, changes in the number of amino acids involved in CEP upon non-covalent binding of EHDP were more evident in the IC isoenzyme. The EC isoenzyme immunoreactivity may correlate with parasite metabolic activity. EHDP and AL decreased the number of CEP-related amino acids on the EC isoenzyme, which may produce a reduction in CEP immunoreactivity. In contrast, BP effect on W6UJ19 increased CEP-related amino acid, potentially enhancing CEP antigenicity and renewing the immune recognition in chronic infections. The EHDP effect could be related to the PPi released from the calcareous body. These PPi would bind the EC enzyme in the Pi site and consequently decrease its antigenicity.

Molecular dynamics simulations showed that the IC isoenzyme presented a greater affinity for both the substrate and Pi, maintaining more stable amino acid interactions throughout the simulation compared to the EC isoenzyme. Although enzymatic activity assays were not performed, the simulation results suggest that the IC isoenzyme is more likely to play a metabolic role in the *E. granulosus* metacestode regarding the MMPBSA results, where substrate affinity is higher in W6UJ19. In contrast, the EC isoenzyme, likely localized in secretion vesicles and/or calcareous corpuscles, appears to participate primarily in host–parasite interaction [15], potentially through protein–protein interactions that facilitate parasite attachment. According to our findings, the quaternary structure of the enzyme remained stable, according to the RMSD results, even when the conformational sites for NAD^+^ or Pi binding were asymmetrically lost by the end of the molecular dynamics simulations. While the quaternary structure of the enzyme is essential for enzymatic activity, it is likely also required for the protein’s non-enzymatic functions, particularly those related to intercellular or intracellular interactions. GAPDH lacks a classical secretion signal peptide, suggesting that its ability to be secreted may depend on interactions with carrier proteins that facilitate its translocation to other cellular compartments [47,48].

Among the BP studied, EHDP reduced cell growth, whereas AL did not produce any measurable effect on in vitro cell behavior, beyond a reduction in ATP levels. This BP acts via different mechanisms: EHDP leads to the accumulation of non-degradable ATP analogs [49], while AL inhibits the protein prenylation pathway. EHDP showed stronger binding to both GAPDH isoenzymes than Pi, although pyrophosphate (PPi) exhibited the highest binding affinity (*p* < 0.005 concerning EHDP, calculated by docking). However, PPi was unable to substitute Pi in the catalytic activity of GAPDH [50]. Unlike PPi, EHDP is not degraded by pyrophosphatases. EHDP also enhanced substrate binding to the IC enzyme and modified the conformational sites for substrate, Pi, and NAD^+^ binding throughout the molecular dynamics simulation, from initiation to completion, suggesting a potential modulation of enzymatic activity. Consequently, we can assume that the GAPDH model with EHDP and without Pi has increased affinity for the substrate in both isoenzymes.

In contrast, AL exhibited lower affinity for GAPDH compared to both the substrate and Pi. EHDP bound more strongly than Pi, and previous studies have reported that phosphonates can interact with GAPDH by binding after Pi removal; the acyl-enzyme intermediate is expected to enhance activity [51]. However, an increase in GAPDH activity may also lead to elevated production of reactive oxygen species, which could overwhelm the cell’s antioxidant defenses and modify the enzyme’s active site, affecting its ability to bind substrate or Pi [42].

One of the known metabolic effects of EHDP is the reduction in glycolysis rate and the promotion of glycogen storage [52] without altering intracellular Pi concentration or uptake [53]. In previous experiments, we observed a decrease in EGPE cell growth after 72 H of EHDP treatment. This selective effect on parasite cells could be attributed to the limited antioxidant capacity of *E. granulosus*, as neither superoxide dismutase nor the thioredoxin system was overexpressed in response to H_2_O_2_ challenge [54]. In fact, EHDP is the BP with the least effect on bone metabolism at a dose used in humans and animals [49,55,56]. Due to its slight effect on bone metabolism and selective effect on EgGAPDH, EHDP, which has been shown to inhibit EGPE cell growth, could be evaluated as an adjuvant treatment for CE localized in bone. As a member of the BP compound family, EHDP exhibits more affinity for bone tissue than for other organs.

Several studies have explored the immunological effects of BP, demonstrating their potential as adjuvant therapies [57] and their ability to promote prolonged antigen persistence within immune cells. However, these studies often did not distinguish whether the immunological modulation was due to BP interference in the mevalonate pathway or a direct effect on the antigenic molecule itself [58].

## 4. Materials and Methods

### 4.1. Ethics Statement and Serum Samples

Serum samples from CE patients were obtained by Dr. Jorge Gentile at the Hospital Municipal Ramón Santamarina, Tandil, Argentina. Dr. Elizabeth Luz Sánchez Romaní, from the Laboratorio de Zoonosis Parasitaria CNSP-INS, Peru, provided sera samples from patients with cysticercosis and fascioliasis. The Ethics Committee of the Universidad Abierta Interamericana, Buenos Aires, Argentina, approved all the protocols and procedures (number 01011) date 28 October 2014, and the ethical protocol approval has continued up to today [12].

### 4.2. Characterization of EgGAPDH

#### 4.2.1. EGPE Cell Culture, Protein Extracts, and Supernatant

EGPE is a cell line derived from the liver bovine metacestode of *E. granulosus* ss/G1 pe. The cell line was obtained in 2008–2010 in our laboratory (Centro de Altos Estudios en Ciencias Humanas y de la Salud, Universidad Abierta Interamericana). Parasite’s pe was digested with papain, and cells cultured in liquid medium 199, antibiotics, β-mercaptoethanol, sodium pyruvate, and 10% filtrated cystic fluid for over one month were discarded because mammalian cells contaminated the parasite cell culture. Afterward, the cystic fluid was changed with 10% inactivated BFS. Cystic colonies were obtained by seeding cells in semisolid 2% agarose medium in 5 mM buffer phosphate and 5mM HEPES (1:1; *v*/*v*), pH 7.5. After 30 min of cell seeding, liquid medium was added, and the plates were cultured in a humid tissue culture incubator 95% air/5% CO_2_. Cells were maintained in our laboratory in liquid nitrogen. Cells used in the experiments corresponded to passages 35 to 40. EGPE cells were cultured, as previously described [11], in medium 199 (Sigma, St. Louis, MO, USA), 1 mM sodium pyruvate (sodium salt, extra pure, Anhedra, Beijing, China), and 78 μg/mL β-mercaptoethanol (Merck, Darmstadt, Germany) at pH 7.9 (37 °C; CO_2_:air; 5/95%). EGPE cell colonies were performed in 2% agarose (20,000 cells/well) [11]. After 20 days of cell growth in a liquid medium, the protein extracts were obtained. EGPE cells were washed five times with DPBS and incubated in lysis buffer (8 mmol/L CHAPS, MP Biomedicals, 10 mmol/L Tris–HCl, pH 8, 2 mmol/L EDTA, 0.1% B-mercaptoethanol, MP Biomedicals, and 1/100 protease inhibitor cocktail, Sigma-Aldrich) at 4 °C for 2 h. The samples were then frozen-thawed three times and spun down at 10,000× *g*. [12].

Cell colonies were grown for 5 days without medium change, supernatants were obtained, and cell debris was removed by centrifuging the supernatants three times (3000 rpm). Finally, samples were aliquoted and stored at −20 °C until use.

#### 4.2.2. Protein Identification

The methodology was previously described by Maglioco et al., 2022 [13]. Briefly, protein fractions were obtained by separating cell extracts based on molecular weight using a Sephacryl S200 HR column (GE Healthcare, Hertfordshire, UK). Eluted protein fractions were identified by absorbance (205–280 nm) using a spectrophotometer (Biowave II+, Biochrome Ltd., Cambridge, UK). Each protein fraction and EGPE supernatant were concentrated through a 3 kDa cut-off membrane concentrator (Pierce, Thermo Scientific, Waltham, MA, USA). Antigenic protein fractions and EGPE supernatant were analyzed by Western blot using a pool of sera from 11 patients with hepatic CE. Reactive protein fractions were subsequently purified by affinity chromatography using Protein G HP SpinTrap columns (GE Healthcare). Sera from patients with cysticercosis and fascioliasis were used as controls. Eluted proteins were concentrated through a 3 kDa cut-off membrane concentrator (Pierce, Thermo Scientific).

A 15% SDS–PAGE was performed to concentrate and clean up the protein extracts before in-gel digestion. Protein bands were visualized by Coomassie blue staining, excised, destained, washed, reduced, alkylated, and then digested in-gel with 100 ng Trypsin (Promega V5111). The resulting peptides were recovered by elution and analyzed by nano LC-MS/MS at the Proteomics Core Facility of the CEQUIBIEM, Faculty of Exact Sciences, University of Buenos Aires/IQUIBICEN CONICET. Protein identification was performed using the *E. granulosus* database from UniProt.org [59], following the procedure described by Maglioco et al., 2022 [13]. Spectrum data was deposited in Project accession: PXD069559 (Table S1). Previously, both GAPDHs were found in ex vivo *E. granulosus metacestode* [46] (PXD067276).

#### 4.2.3. EgGAPDH Genomic Sequence

*E. granulosus* DNA was extracted from a G1 genotype metacestode isolated from the liver of a cow obtained at a local slaughterhouse in Buenos Aires, Argentina (provided by Dr. Tatiana Aronowicz, SENASA). Primers for amplification of the complete GAPDH gene sequence were designed using the Primer-Blast tool available on the NCBI website [60]. The fragment size ranged from ≈800–1000 bp.

For W6UJ19 (IC), the forward primer was GAGGCCCAACACCGGAATTA and the reverse CCACGGGTTTCAGTAATGAGC (Tm 54.36 °C; extension time: 1 min 3 s). For W6V1T8 (EC), the forward primer was TCGAGAAGGCCTCGGTAAGA and the reverse primer was TCCAGCGGGAGCCTTAATGA (Tm 54 °C; extension time: 50 s). Primers were purchased from Gene Biotech SRL, Buenos Aires, Argentina. Each reaction tube contained: 1.5 mM MgCl2 (5x, Colorless GoTaq^®^ Reaction Buffer, Promega, Madison, WI, USA), 0.2 mM of dNTPs mix (dGTP, dCTP, dTTP and dATP, Promega, Madison, WI, USA), 1 µM forward primer, 1 µM reverse primer, 1.25 units of DNA polymerase (GoTaq^®^ polymerase, Promega, Madison, WI, USA), 18 ng of DNA template and nuclease-free water up to 25 µL (ultrapure, PB-L, Productos Bio-lógicos). DNA amplification was performed with an initial denaturation step at 95 °C for 2 min, followed by 35 cycles of denaturation at 95 °C for 1 min, primer annealing at the respective temperature for each primer pair for 1 min, and extension at 72 °C for a time determined by the expected product size. A final extension step was carried out at 72 °C for 5 min. Reactions were conducted using a Mastercycler gradient thermocycler (Eppendorf, Hamburg, Germany). Then, 17.5 µL of each product was analyzed by agarose gel electrophoresis (LE molecular biology grade, PB-L, Productos Bio-Lógicos, Buenos Aires, Argentina) using a 100–1100 bp ladder (Dongsheng Biotech Co., Ltd., Guangzhou, China) as a size marker. DNA bands were excised and purified using the Wizard^®^ SV Gel and PCR Clean Up System (Promega Co., USA). Purified PCR products were sequenced at the CEDIE “Dr César Bergadá (CONICET- Hospital de Niños Ricardo Gutiérrez, Buenos Aires, Argentina). The resulting sequences were analyzed using BLAST and manual alignment. Amino acid sequences corresponding to predicted epitopes were identified from the full-length protein sequences retrieved from the BLAST results.

#### 4.2.4. Amino Acid Sequence, Protein Structure, and Physicochemical Analysis

**Figure 8:**
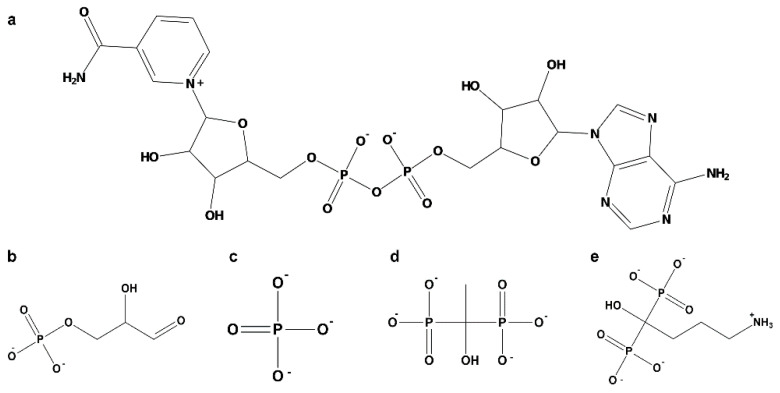
Two-dimensional chemical structure of the studied ligands in the ionization state used in computational simulations. (**a**) NAD^+^, (**b**) Glyceraldehyde-3-phosphate (G3P), (**c**) inorganic phosphate (Pi), (**d**) etidronate (EHDP), (**e**) alendronate (AL).

The complete amino acid sequences of both GAPDHs were obtained from UniProt. Analysis of differences in GAPDH protein sequences was carried out using BLAST-P, using the complete sequence of each isoenzyme and a non-redundant database and organism as inputs. Physicochemical properties were analyzed using the ProtParam tool from the Expasy server [61]. Secondary structure prediction of *E. granulosus* GAPDH was conducted using SOPMA [62]. GAPDH domains were predicted using Pfam [63], NCBI [64], and Interpro [65]. Modeling of the tertiary structure was performed using Phyre2 normal mode [66]. The protein models obtained by Phyre2 have been superposed to AlphaFold3 models [67], and the root mean square deviation (RMSD) between the two models was evaluated. Model quality was assessed by ProSA-web [68]. To assemble GAPDH monomers into a quaternary structure, we modeled the 222 symmetry homotetramer (biological assembly) using *Leishmania mexicana* GAPDH (PDB code: 1I32) as a template. The model of *Acropora millepora* (PDB code: 6PX2) was used to map the binding sites for substrates Pi, and NAD^+^. The protonation and tautomeric states of titratable residues, and the orientation of hydrogen atoms, were assigned using the Protonate3D module implemented in MOE (MOE 2022.02). This procedure simultaneously optimizes ionization, tautomeric, and hydrogen-bonding states based on a free-energy model including Van der Waals, Coulomb, solvation, and titration terms [69]. Calculations were performed at pH 7 and a saline concentration of 0.1 M. Histidine residues were protonated at Nε2 (HIE) unless hydrogen bonding suggested an alternative tautomeric state. Ligands (G3P, Pi, EHDP, and AL) were parameterized in their predominant ionization states at neutral pH: G3P as a dianion, EHDP as a tetraanionic species, and Pi and AL as trianionic species. The resulting protonation states were used to generate the fully protonated structures for subsequent energy minimization, docking, and molecular dynamics simulations, and are shown in Figure 8.

### 4.3. Molecular Dynamics Simulation

#### Native Interactions of GAPD

The GAPDH molecular structures were validated through molecular dynamics simulation using the Nanoscale Molecular Dynamics software (version 3.0, NAMD2) [70]. GAPDH was solvated with explicit solvent by using the TIP3 water model. For W6UJ19, the water box size was 114.83 × 122.65 × 107.08 Å (x, y, z), containing 40,077 water molecules. For W6V1T8, the water box was 116.29 × 117.06 × 110.21 Å (x, y, z), containing 40,085 water molecules. System neutralization was performed to reflect the distinct localization of both enzymes; KCl was used for the IC (W6UJ19) and NaCl for the EC isoenzyme (W6V1T8). The ionic concentration was 0.15 mol/L in both cases. All simulations were performed under the periodic boundary conditions. The counterion content was 112 Cl^−^ and 128 K^+^ atoms and 112 Cl^−^ and 129 Na^+^, for W6UJ19 and W6V1T8, respectively. All molecular dynamics simulations were performed using the CHARMM36 force field under NPT Ensemble (Isothermal-Isobaric) conditions: constant particle number, pressure (1 atm), and temperature (300 K). The simulation protocols involved 20,000 steps for minimization by the conjugate gradient method plus 144,000 steps (0.288 ns) of heating from 60 to 300 K, maintaining the absolute temperature at 300 K for the following steps. Afterward, 500,000 steps (1 ns) were performed to equilibrate with restrictions in the backbone, and finally, a 100 ns unrestrained trajectory was obtained for production. The time of each step was 2 fs. The potential energy throughout the simulation was monitored to confirm the thermodynamic equilibrium of each system. The root mean square deviation (RMSD) and root mean square fluctuation (RMSF) were calculated. Structures were analyzed and visualized with Visual Molecular Dynamics (version 1.9.3) [71] and Molecular Operating Environment (MOE 2022.20) [72].

- Free energy binding estimation.

The free energy binding between the protein chains and the ligand molecules was estimated by the MMPBSA method using the last 100 frames of each molecular dynamic simulation with the VMD CAFE1.0 tool and the APBS program. For each estimation, the entire molecular system was defined as the complex, the target molecule as the ligand, and all remaining molecules in the system (excluding the ligand) as the receptor.

### 4.4. Docking and Enzymatic Dynamic Interaction with BP

#### 4.4.1. Docking

Molecular docking simulations of the natural substrate of GAPDH, glyceralde-hyde-3-phosphate (G3P), Pi, and two BPs, EHDP (PubChem: CID3305) and AL (PubChem: CID2088), were performed using the Dock module of MOE 2022.20. The catalytic site corresponding to the substrate-binding region of each GAPDH enzyme with NAD^+^ positioned in its corresponding binding site was defined by selecting residues within a sphere of 4.5 Å radius centered on the catalytic cysteine.

Ligand conformations were generated by systematic bond rotations prior to placement. Poses were generated using the Alpha Triangle placement method, which aligns triplets of ligand atoms with triplets of α-sphere centers within the defined binding site. Each ligand yielded up to ten poses, initially scored with the London ΔG function and subsequently refined through energy minimization using the GBVI/WSA ΔG scoring scheme. The top-ranked pose for each ligand was selected for subsequent molecular dynamics simulations. Differences among docking scores were analyzed using the Student *t*-test, considering that each enzyme has 4 subunits; each subunit was considered a single unit for analysis.

#### 4.4.2. Molecular Dynamics Interaction

Protein-ligand complexes were prepared for molecular dynamics simulation using MOE software (version 2024.06) and the QwikMD tool [73] available in VMD 1.9.3, as previously described. All molecular dynamics simulations were performed on the homotetrameric quaternary structures of both GAPDH isoenzymes, each composed of four identical subunits arranged in 222 symmetry, as described for other GAPDH enzymes. The tetrameric assemblies were generated based on the *L. mexicana* GAPDH template (PDB ID: 1I32) and validated for structural stability during the simulations. Ligand three-dimensional geometries were manually built and optimized in MOE 2022.20. Initial geometry optimization was performed using the MMFF94x force field to refine bond lengths, angles, and torsions. For molecular dynamics simulations, the ligands were parameterized using the CHARMM General Force Field (CGenFF), ensuring full compatibility with the CHARMM36 force field employed for the protein systems. Each optimized ligand structure was visually inspected to confirm correct bond orders, protonation states, and stereochemistry prior to docking and simulation.

A 100 ns production simulation was performed without backbone restraints. Molecular dynamics settings were the same as outlined in Section Native Interactions of GAPD, with modifications in the number of water molecules and ions for each system. For the W6UJ19-EHDP complex, the solvation box contained 40,078 water molecules along with 112 Cl^−^ and 116 K^+^ ions. For the W6UJ19-AL complex, the system included 40,081 water molecules and 116 Cl^−^, with 112 K+ ions. In the W6V1T8-EHDP system, 40,084 water molecules were used, along with 112 Cl^−^ and 116 Na^+^ ions. The W6V1T8-AL system contained 39,980 water molecules and 116 Cl^−^ with 112 Na^+^ ions. All simulations were executed remotely using NAMD2 on high-performance computing equipment at the Department of Experimentation and Theory of the Structure of Matter and its Applications (DETEMA, Facultad de Química, Universidad de la República, Montevideo, Uruguay). Hardware specifications included: AMD Ryzen 9 5950X processor, RAM 64 GB, RTX 3080 TI GPU, running Ubuntu 22.04.1.

### 4.5. Searching for Epitopes

#### 4.5.1. B-Cell LEP

LEP prediction was conducted on GAPDH sequences from *E. granulosus*, *T. solium*, *F. hepatica*, *E. multilocularis, Bos taurus*, *Ovis aries*, *Homo sapiens*, and *L. mexicana* using eight epitope prediction programs. The B-cell LEP for each GAPDH were selected from ABCpred (with a score above 0.85) [74], identified in regions of at least five adjacent amino acids by Bepipred Linear Epitope Prediction 2.0 (threshold: 0.5) or by Bepipred Linear Epitope Prediction (threshold: 0.35), and by at least three of the following software: Chou & Fasman Beta-Turn Prediction (threshold indicated by the server for each protein sequence), Emini Surface Accessibility Prediction (threshold: 1.0), Karplus & Schulz Flexibility Prediction (threshold: 1.0), Kolaskar & Tongaonkar Antigenicity (threshold indicated by the server for each protein sequence), and Parker Hydrophilicity Prediction (threshold indicated by the server for each protein sequence) in IEDB (Immune Epitope Database and Analysis Resource) [75]. Post-translational modifications were predicted using MusiteDeep [76] (cut off 0.50) and DeepNitro [77] (cut off medium score).

#### 4.5.2. B-Cell CEP and BP

B-cell CEP for each GAPDH protein was predicted using DiscoTope 2.0, applying a threshold of −3.7 (sensitivity = 0.47 and specificity = 0.75). Predictions were based on the PDB structures of each GAPDH system at both the beginning and end of the molecular dynamics simulation, including IC and EC isoenzymes under control conditions and after binding with EHDP and AL.

## 5. Conclusions

We performed the first in silico studies on *E. granulosus* GAPDH, an essential enzyme of the glycolytic pathway involved in host–parasite interactions, recognized by sera from CE patients. Based on the predicted LEP in two GAPDH isoenzymes, the IC isoenzyme (W6UJ19) emerged as the best candidate for the design of multiepitope constructs with potential applications in CE diagnosis, and the ratio between the IC and EC isoenzymes will be useful to evaluate treatment success. Moreover, the CEP modification by EHDP on W6UJ19 could enhance immunological surveillance, although it may decrease antigenicity of W6V1T8.

Molecular dynamics simulations suggest that the IC isoenzyme has a greater probability of being modulated by BP compared to the extracellular (EC) isoenzyme (W6V1T8), supporting its potential as a pharmacological target. Simulations examining the interaction of two BPs, EHDP and AL, with both isoenzymes showed that EHDP (a first-generation BP) exhibits stronger binding affinity than AL. This finding aligns with our previous in vitro results using EGPE. Indeed, EHDP, due to its limited effect on bone metabolism and its potential antiparasitic effect, could be a candidate for translational research in infections caused by *E. granulosus* in intermediate hosts. Notably, EHDP shares a structural similarity with PPi, suggesting that endogenous PPi could potentially interact with GAPDH and modulate its enzymatic activity or interfere with EHDP’s effect; however, only PPi is metabolized by endogenous pyrophosphatase. The simulations also provided insights into molecular stability and substrate interactions affected or unaffected by BP binding. Furthermore, we demonstrated how CEP could be altered upon binding of those interactive compounds.

## Figures and Tables

**Table 1:** Comparative GAPDH physicochemical properties from different species.

GAPDH Specie (UniProt)	Amino Acid Number / M.W.	Charge	Isoelectric Point	Protein Instability	Aliphatic Index	Gravy Index
***Echinococcus granulosus* (W6UJ19)**	338 / 36.6	+1 (R + K: 37; N + E: 36)	7.56	24.45	86.55	−0.100
***Echinococcus granulosus* (W6V1T8)**	336 / 36.1	+3 (R + K: 35; N + E: 32)	8.44	21.82	85.89	0.004
***Echinococcus multilocularis* (Q27652)**	336 / 36.4	+7 (R + K: 39; N + E: 32)	9.02	27.10	84.40	−0.090
***Taenia solium* (A8R8Q4)**	336 / 36.3	+3 (R + K: 35; N + E: 32)	8.44	19.73	86.19	−0.029
***Fasciola hepatica* (A0A068LJN3)**	338 / 36.9	0 (R + K: 37; N + E: 37)	7.10	25.15	84.76	−0.109
***Leishmania mexicana*** **(Q27890)**	361 / 39.0	+7 (R + K: 42; N + E: 35)	9.05	27.63	83.96	−0.115
***Homo sapiens* (P04406)**	335 / 36.0	+3 (R + K: 36; N + E: 33)	8.57	15.04	85.55	−0.112
***Bos taurus* (P10096)**	333 / 35.9	+3 (R + K: 36; N + E: 33)	8.51	17.70	84.89	−0.078
***Ovis aries* (Q28554)**	322 / 34.7	+1 (R + K: 34; N + E: 33)	7.83	17.38	85.09	−0.091

**Table 2:** Glyceraldehyde-3-phosphate dehydrogenase LEP and the post-translational modifications.

GAPDH Isoenzyme	LEP	Residue	Predicted Modification(s)
**W6UJ19**	A82-EAIPWDKDGVYYVV-E97	W87	Nitration
		K89	Ubiquitination
	D88-KDGVYYVVESTGVN-T103	K89	Ubiquitination
	S123-APSKDAPTFVVGVN-L138	-	-
	K140-YDPSMTIVSNASCT-T155	S149	Phosphorylation
		N150	Glycosylation
		S152	Phosphorylation
		T154	Phosphorylation
	Q186-KLVDGPNPKGWRDG-R201	K195	Acetylation Methylation
	D282-VVSMDFRTSTASST-F297	S285	Phosphorylation
	F288-RTSTASSTFDANAG-I303	-	-
**W6V1T8**	K138-YDPSMKVVSNASCT-T153	S147	Phosphorylation
		N148	Glycosylation
		C151	Nitrosylation
		S150	Phosphorylation
		T152	Phosphorylation
		T153	Phosphorylation
	F286-LSTTCSSTFDARAG-I301	-	-

**Table 3:** Total potential energy of both EgGAPDH isoenzymes during molecular dynamics simulation after equilibration step (100 ns of unrestrained production), partitioned into electrostatic and Van der Waals contributions; all the values are in kcal/mol.

GAPDH		Electrostatic		Van der Waals		Total Potential Energy	
		Mean	SD	Mean	SD	Mean	SD
**W6UJ19**	MD	−498,905.11	484.6043	35,705.02	302.8204	−433,534.22	357.6353
**W6V1T8**	MD	−500,354.14	483.1283	35,967.32	303.1194	−435,622.22	344.5178

**Table 4:** ∆G of binding estimated for Pi and EHDP to IC and EC EgGAPDH in each simulation system.

Ligand	∆G of Binding (kcal/mol)	
	IC W6UJ19	IC W6V1T8
Pi (system GAPDH: Pi, G3P, NAD^+^)	−2.136 ± 1.366	−3.477 ± 0.098
EHDP (system GAPDH: EHDP, G3P, NAD^+^)	−4.413 ± 0.313	−4.363 ± 0.331

All measured averages were analyzed using Student’s *t*-test, resulting in *p* < 0.05, confirming that the differences were statistically significant.

**Table 5:** ∆G of binding estimated for NAD^+^ to IC and EC EgGAPDH in control and AL in each simulation system.

Ligand	∆G of Binding (kcal/mol)	
	IC W6UJ19	EC W6V1T8
NAD^+^ (system GAPDH: Pi, G3P, NAD^+^)	−7.727 ± 0.674	−9.274 ± 0.419
NAD^+^ (system GAPDH: AL, NAD^+^)	−9.418 ± 0.644	−10.376 ± 0.771

In consequence, the substitution of AL by G3P and Pi resulted in an energy interaction significantly increased for the NAD^+^ molecule (Student’s *t*-test *p* < 0.05).

**Table 6:** Binding free energy (∆G, kcal/mol) estimated by the MMPBSA method for EgGAPDH isoenzymes in the presence and absence of BP.

MMPBSA of EgGAPDH With and Without BP							
CONTROL		W6UJ19			W6V1T8		
Subunit	Ligands	Mean	±sd	±CI 95%	Mean	±sd	±CI 95%
A	G3P	−10.6968	5.1229	1.005	−4.724	3.2619	0.64
B		15.4506	5.1449	1.01	3.9659	4.4619	0.875
C		16.4556	4.8065	0.945	44.9831	7.6067	1.49
D		24.028	3.8583	0.755	6.2637	4.5538	0.895
A	NAD^+^	22.4688	6.7112	1.315	6.6577	5.4992	1.08
B		32.8592	7.3992	1.45	−4.0176	3.6996	0.725
C		13.5946	4.3631	0.855	23.7809	5.5271	1.08
D		6.0962	4.2739	0.835	−0.61	2.8136	0.55
A	Pi	29.6458	6.7445	1.325	6.7074	3.0	0.59
B		39.1984	7.6647	1.5	1.2245	10.312	2.025
C		22.235	6.1243	1.205	45.3445	5.9314	1.165
D		37.2889	7.0652	1.385	2.1399	10.741	2.11
**EHDP**							
A	G3P	−16.9965	4.7622	0.935	−7.9112	3.9841	0.78
B		5.2706	3.8102	0.75	−7.5877	3.8438	0.755
C		−5.1953	7.1869	1.405	−7.9317	3.1745	0.62
D		−9.1029	5.5589	1.09	−14.587	4.1819	0.82
A	NAD^+^	2.4725	10.0795	1.975	1.7223	5.9318	1.16
B		7.3854	4.7754	0.935	6.221	5.0763	0.995
C		27.6537	8.7531	1.715	−4.6031	3.9027	0.765
D		28.9197	5.1906	1.02	4.4928	4.5107	0.885
A	EHDP	−6.6067	3.3539	0.655	−4.0724	3.0422	0.595
B		−7.1715	2.9301	0.575	0.5011	3.9765	0.78
C		0.815	5.1088	1.005	−11.8148	3.0683	0.605
D		2.0767	4.4194	0.865	−1.3616	3.2844	1.29
**AL**							
A	NAD^+^	24.1947	7.3184	1.435	9.1407	6.0582	1.19
B		2.1941	4.4401	0.87	8.8646	5.4337	1.065
C		−3.7258	3.5832	0.705	5.2785	4.2828	0.84
D		−3.0283	2.003	0.39	−3.2583	3.9806	0.78
A	AL	19.1598	3.9525	0.77	7.624	3.2452	0.635
B		−1.2968	2.2008	0.43	0.3786	2.3406	0.46
C		4.3972	3.5114	0.685	6.9541	3.3836	0.665
D		8.2511	4.1911	0.82	−1.8823	13.8439	2.715

**Table 7:** List of amino acids interacting with ligands in the IC EgGAPDH in each simulation system. In bold: amino acid that makes frequent contacts with ligands.

W6UJ19 Control	Initial			Final		
	G3P	Pi	NAD^+^	G3P	Pi	NAD^+^
Subunit A	**R235**(2)	T154 S152 T212	G12(2) N318(2) **S123**(2)	**R235**(3) His180(2) T183 C153	K195(3) R235(2)	**S123**
Subunit B	C153 **H180** **T183**	C153(2) T154 H180	G12 **R13** **D35** N318(2)	R235(2) **H180(2)** **T183**	R235(3) K195(2)	**R13** **D35** D190
Subunit C	R235(2)	T154	R13 H180 EA319 **K187**	T212(2) G213	R235(2) H180(2)	E319(2) D190 N193 **K187**
Subunit D	H180	T154(2) H180 G213	**D35** S98 C153	C153	K195 R235	**D35** T99
**W6UJ19** EHDP	Initial			Final		
	G3P	EHDP	NAD^+^	G3P	EHDP	NAD^+^
Subunit A	R235(2)	T154(2) Q211 T212 G213 **R235**	G12(2) S98 S123(2) N318(2)	T183 H180(3)	Q211 **R235**	D337
Subunit B	**C153** H180 T183	C153 T154 H180	G12 R13 D35 N318(2)	**C153**	D199(2) Q211(2) P209	K195
Subunit C	R235(2)	T154(2) **T212**	R13 H180 K187 **E319**	H180(2)	**T212**	P192 **E319**
Subunit D	**C153**	T154 **Q211** **T212**(2) G213	**D35** S98 C153	S123 **C153**(2) H180(2) T183 N318	**Q211** **T212** R235	**D35** E319
**W6UJ19** AL	Initial			Final		
	AL		NAD^+^	AL		NAD^+^
Subunit A	T154(2) Q211 T212(2) G213 R235		G12(2) S98 S123(2) N318(2)	S123		A184 E319 K187 D190
Subunit B	C153 T154 H180		G12 R13 D35 N318(2)	I38		-
Subunit C	T212 T154(2)		R13 H180 **K187** E319	S126 D128(2) A217		T103(2) I122 **K187**
Subunit D	T154 Q211 T212(2) G213		D35 S98 C153	A124 S149 S152		-

**Table 8:** List of amino acids interacting with ligands in the EC EgGAPDH in each simulation system. In bold: amino acid that makes frequent contacts with ligands.

W6V1T8 Control	Initial			Final		
	G3P	Pi	NAD^+^	G3P	Pi	NAD^+^
Subunit A	T181 C151 **R233(2)**	S150 C151(2) T152(2)	**R12** I13 S97 **S121(2)** N316(2)	**R233(3)** K193(2)	-	**R12(2)** D34(2) T98 **S121** E317
Subunit B	C151 **R233**	C151 T152(2)	R12(2) **D34(2)** S121 N316(2)	**R233(2)** K193(2)	-	G11 **D34(2)**
Subunit C	C151	S150 C151(2) T152 T210	R12 I13 S97(2) Y320	R233	R233(2) K233(3)	T181 A182 P237
Subunit D	Cys151(2)	S150 C151 T152(2)	G11(2) R12 I13	N101 K193 R233(3)	-	-
**W6V1T8** EHDP	Initial			Final		
	G3P	EHDP	NAD^+^	G3P	EHDP	NAD^+^
Subunit A	C151 T181 **R233**	T152(2) T210	G11 **R12** I13 S121(2) N316(2)	K193(2) **R233(2)**	-	F10 **R12** D34(2) F36
Subunit B	S150 C151 **R233**	T152(2)	**R12**(2) **D34** E78 **S121** N316(2) E317	K193(2) **R233(2)**	-	F10 **R12** **D34**(2) **S121**
Subunit C	C151	C151(2) T152 H178	**R12** I13 P35 E78 S97(2) Y320	K193(2)	G99	G11 **R12** D34(2)
Subunit D	C151 T183	C151 **T152**	G11 R12 I13 P123(2)	K193 R233(2)	**T152** T210	P35 S97 T98 S121 **P123** T181 T183 K192
**W6V1T8** AL	Initial			Final		
	AL		NAD^+^	AL		NAD^+^
Subunit A	C151		G11 R12 I13 S121(2) D316(2)	T181		F10 D34(2) F36
Subunit B	C151		**R12** **I13** **D34** E78 C151 E317	D165 N166		G11 **R12**(2) **I13** **D34**(2)
Subunit C	C151(2) E317		**R12** I13 S97(2) Y320	N316		**R12** D34(2) E317
Subunit D	C151(2) H178		G11(2) R12 I13 D34	-		P190 K185 K192

**Table 9:** CEPs, post-translational modifications, and changes induced by BP on EgGAPDH W6UJ19 intracellular and W6V1T8 extracellular isoenzymes. System simulations: 1—control: Pi, G3P, and NAD^+^. 2—EHDP: EHDP, G3P, and NAD^+^. 3—AL: AL and NAD^+^. Post-translational modifications: -CH_3_: methylation; -CH_3_CO: acetylation; Ph: phosphorylation; and Ubiq: ubiquitination.

Isoenzyme	System	Chain A	Chain B	Chain C	Chain D
W6UJ19	Control	K63, D64, G65, K66, L80, K89, A106, K107, G109, A110, L112, K113, N114, N115, S144, N193, P194, K195	L80, N81, E83, A108, G109, A110, H111, L112, K113, N114, N115, S144, N193, P194	E79, L80, N81, A82, E83, K89, K113, S144, T185, N193, P194, K195, G200, D337	I38, D39, D64, N81, A82, A106, G109, K113, S144, P192, N193, P194
	AL	D64, G65, K66, L80, N81, K89, A106, K107, G109, A110, L112, K113, N114, N115, S144, P192, N193, P194, K195	L80, N81, A82, A84, I85, P86, W87, D88, K89, D90, A108, G109, A110, H111, L112, K113, N114, S144, N193, P194, K195	L80, N81, A82, E83, K89, H111, S144, P192, N193, P194, K195, G200	N81, A106, A110, K113, N114, N115, S144, T185, N193, P194, K195
	EHDP	D39, K63, D64, G65, K66, K107, G109, A110, K113, N114, N115, S144, N193, P194, K195, G196, D337	T41, L112, K113, S144, P192, N193, P194, K195	L80, N81, A82, E83, A106, S144, N193, P194, K195	N81, K107, A110, H111, K113, N114, N115, S144, P194, K195, G196, G200
Post-translational modifications	-CH_3_/-CH_3_CO	K66 and K195	K195	K195	K195
	Ubiq	K89	K89	K89	-
	Ph	-	-	-	T185
W6V1T8	Control	S142, K192, K193	P141, S142, K192, K193	K192, D335	S142, K192
	AL	P141, S142, K192	S142, K192	S142	P141, K193
	EHDP	K192	S142, K192	D38, K138, K192, K193	P190, K192
Post-translational modifications	-CH_3_	K192	K192	K192	K192
	-CH_3_/-CH_3_CO	K193	K193	K193	K193

## Data Availability

The original contributions presented in this study are included in the article/Supplementary Material. Further inquiries can be directed to the corresponding authors.

## Supplementary Materials

The following supporting information can be downloaded at https://www.mdpi.com/article/10.3390/ijms262110622/s1.
