# Supplementary material for: First In Silico Study of Two Echinococcus granulosus Glyceraldehyde-3-Phosphate Dehydrogenase Isoenzymes Recognized by Liver Cystic Echinococcosis Human Sera
Source: Int J Mol Sci. 2025 Oct 31;26(21):10622. doi: 10.3390/ijms262110622 (PMC12607693; doi:10.3390/ijms262110622)
Supplement: Supplementary file 1 [file ijms-26-10622-s001.zip › Captions.pdf]

International Journal of
Molecular Sciences

MDPI

Figure S1: GAPDH PCR: S1a and b PCR of IC W6UJ19 show blast result coding sequence obtained from the PCR product. **S1a** shows 86.74% identity and 99% coverage with the reference sequence XM_024492989.1. The forward primer was the oligonucleotide 5′-GAGGCCCAACACCGGAATTA-3′. **S1b:** The reverse coding obtained from the PCR product shows 93.53% identity and 78% coverage with the reference sequence XM_024492989.1. The following oligonucleotide 5′-CCACGGGTTTCAGTAATGAGC-3′ was employed.

S1c and d PCR of EC W6V1T8: S1c: It shows blast result of the forward coding sequence. PCR product shows 81.31% identity and 72% coverage with the reference sequence XM_024494574.1. The oligonucleotide 5′-TCGAGAAGGCCTCGGTAAGA-3′ was employed. **S1d:** Blast result of the reverse coding sequence obtained from the PCR product shows 98.54% identity and 83% coverage with the reference sequence XM_024494574.1. The following oligonucleotide 5′- TCCAGCGGGAGCCTTAATGA-3′ was employed.

Figure S2: Tertiary structure stability of EgGAPDH monomers. In a), model quality assessment of Phyre2 models using PROSA-web. Z scores are shown in blue for NMR structures and light blue for X-ray structures. In b), structural stability over 100 ns of molecular dynamics production simulation was evaluated by RMSD.

Figure *S3:* Stability of energy in molecular dynamics simulations: Energy in kcal/mol for all GAPDH system vs time in ns. Green is for electrostatics, red for Van Der Waals and blue for total Potential energy. A, b and c show IC W6UJ19: a, control; b, EHDP and c, AL. D, e and f show EC W6V1T8: d, control; e, EHDP, and f, AL

Figure S4: Amino acid interactions in IC W6UJ19 in molecular dynamics simulation**:** Amino acids involved in binding to G3P, NAD^+ and Pi from the beginning and the end after 100 ns simulation. Interactions are mapped across the four monomers of the control tetrameric structure.

Figure S5: Amino acid interactions in EC W6V1T8 in molecular dynamics simulation: Amino acids involved in binding to G3P, NAD^+ and Pi from the beginning and the end after 100 ns simulation. Interactions are mapped across the four monomers of the control tetrameric structure.

Figure S6: RMSD values of GAPDH with the addition of BP; Root mean square deviation (RMSD) profiles over 100 ns of molecular dynamics simulation, showing the structural stability of IC W6UJ19 (a) and EC W6V1T8 (b). The control system is the enzyme with substrate, Pi and NAD^+ without BP. EHDP system is the isoenzyme with EHDP, substrate and NAD^+ and AL system is isoenzyme with AL and NAD^+.

Figure S7: Amino acid interactions in IC W6UJ19-EHDP in molecular dynamics simulation: Interaction was studied from the beginning to the end of the molecular dynamics simulation. Residues involved in interaction with G3P, EHDP and NAD^+ are shown.

Figure S8: Amino acid interactions in EC W6V1T8 -EHDP in molecular dynamics simulation: Interaction was studied from the beginning to the end of the molecular dynamics simulation. Residues involved in interaction with G3P, EHDP and NAD^+ are shown

Figure S9: Amino acid interactions in IC W6UJ19-AL in molecular dynamics simulation: Interaction was studied from the beginning to the end of the molecular dynamics simulation. Residues involved in interaction with AL and NAD^+ are shown

Figure S10: Amino acid interactions in EC-W6V1T8-AL in molecular dynamics simulation: Interaction was studied from the beginning to the end of the molecular dynamics simulation. Residues involved in interaction with AL and NAD^+ are shown.

Table S1: Proteomic data (PXD069559)

Table S2: Sites of cofactors and substrate in GAPDH of different species.
Table S3: *Echinococcus granulosus* GAPDH ligands interactions.
