# Supplementary material for: First In Silico Study of Two Echinococcus granulosus Glyceraldehyde-3-Phosphate Dehydrogenase Isoenzymes Recognized by Liver Cystic Echinococcosis Human Sera
Source: Int J Mol Sci. 2025 Oct 31;26(21):10622. doi: 10.3390/ijms262110622 (PMC12607693; doi:10.3390/ijms262110622)
Supplement: Supplementary file 1 [file ijms-26-10622-s001.zip › Table S2.pdf]

Table S2: sites of binding

GAPDH<br>Specie<br>UNIPROT	Substrate	NAD+	Pi<br>conformational site	Glyceraldehyde-3-phosphate phosphate	1,3-bisphosphoglycerate phosphate
Echinococcus granulosus<br>W6UJ19	C153	S123, N318	S152, T154, T212, G213	T183, T185, R235	R235, T183, T185
Echinococcus granulosus<br>W6V1T8	C151	S121, N316	S150, T152, T210, G211	T183, T185 R235	R233, T181, T183
Echinococcus multilocularis<br>*Q27652*	C151	S121, N316	S150, T152, T210, G211	T181, T183 R233	R23, T181, T183
Taenia solium<br>*A8R8Q4*	C151	S121, N316	S150, T152, T210, G211	T181, T183, R233	R233, T181, T183
Fasciola hepatica<br>*A0A068LJN3*	C153	S123, N317	S152, T154, T212, T213	T182, T171, R221	R234, D215, R217
Leishmania mexicana<br>Q27890	C167	S135, N336	S168, T167, T227, G228	T198, T200, R250	R250, T198, T200
Homo sapiens<br>P04406	C152	S122, N316	S151, T153, T211, G212	T182, T184, R234	R234, T254, T256
Bos Taurus<br>P10096	C150	S120, N314	S149, T151, T209. G210	T180, T182, R235	R232, T180, T182
Ovis aries<br>*Q28554*	C139	S109, N303	S138, T140, T198, G199	T169, T171, R221	R221, T169, T171
