# Supplementary material for: First In Silico Study of Two Echinococcus granulosus Glyceraldehyde-3-Phosphate Dehydrogenase Isoenzymes Recognized by Liver Cystic Echinococcosis Human Sera
Source: Int J Mol Sci. 2025 Oct 31;26(21):10622. doi: 10.3390/ijms262110622 (PMC12607693; doi:10.3390/ijms262110622)
Supplement: Supplementary file 1 [file ijms-26-10622-s001.zip › Table S3.pdf]

Suppl Table S3- Ligands interactions

W6UJ19 CONTROL	Initio			Final		
Subunit	G3P	Pi	NAD+	G3P	Pi	NAD^+
A	R35	T154<br>S152<br>T212	G12<br>N318<br>S123	R235(3)<br>H180<br>T183<br>C153	K195(3)<br>R235(2)	S123
B	C153<br>H180<br>T183	C153<br>T154<br>H180	G12<br>R13<br>D35<br>N31	R235<br>H180<br>T183	R23<br>K195	R13<br>D35<br>D190
C	R235(2)	T154	R13<br>H180<br>EA319<br>K187	T212<br>G213	R235<br>H180	E319<br>D190<br>N193<br>K187
D	H180	T154<br>H180<br>G213	D35<br>S98<br>C153	C153	K195<br>R235	D35<br>T99
**W6UJ19 EHDP**	Initio			Final		
Subunit	G3P	EHDP	NAD+	G3P	EHDP	NAD+
A	R235(2)	T154<br>Q211<br>T212<br>G213<br>R235	G12<br>S98<br>S123<br>N318	T183<br>H180	Q211<br>R235	D337
B	C153<br>H180<br>T183	C153<br>T154<br>H180	G12<br>R13<br>D35<br>N318	C153	D199<br>Q211<br>P209	K195
C	R235	T154<br>T212	R13<br>H180<br>K187<br>E319	H180	T212	P192<br>E319
D	C53	T154<br>Q211<br>T212<br>G213	D35<br>S98<br>C153	S123<br>C153<br>H18<br>T183<br>N318	Q211<br>T212<br>R235	D35<br>E319
**W6UJ19 AL**	Initio			Final		
Subunit	AL		NAD+	AL		NAD+
A	T154<br>Q211<br>T212<br>G213<br>R235		G12<br>S98<br>S123<br>N318	S123		A184<br>E319<br>K187<br>D190
B	C153<br>T154<br>H180		G12<br>R13<br>D35<br>N318	I38		-
C	T212		R13	S126		T103

	T154	H180<br>K187<br>E319	D128<br>A217	I122<br>K187
D	T154<br>Q211<br>T2128<br>G213	D35<br>S98<br>C153	A124<br>S149<br>S152	-

**W6V1T8 CONTROL**	Initio			Final		
Subunit	G3P	Pi	NAD+	G3P	Pi	NAD+
A	T181<br>C151<br>R233	S150<br>C151<br>T152	R12<br>I13<br>S97<br>S121<br>N316	R233<br>K193	-	R12<br>D34<br>T98<br>S121<br>E317
B	C151<br>R233	C151<br>T152	R12<br>D34<br>S121<br>N316	R233<br>K193	-	G11<br>D34
C	C151	S150<br>C151<br>T152<br>T210	R12<br>I13<br>S97<br>Y320	R233	R233<br>K233	T181<br>A182<br>P237
D	Cys151	S150<br>C151<br>T152	G11<br>R12<br>I13	N101<br>K193<br>R233	-	-

**W6V1T8 EHDP**	Initio			Final		
Subunit	G3P	EHDP	NAD+	G3P	EHDP	NAD+
A	C151<br>T181<br>R233	T152<br>T210	G11<br>R12<br>I13<br>S121<br>N316	K193<br>R233	-	F10<br>R12<br>D34<br>F36
B	S150<br>C151<br>R233	T152	R12<br>D34<br>E78<br>S121<br>N316<br>E317	K193<br>R233	-	F10<br>R12<br>D34<br>S121
C	C151	C151<br>T152<br>H178	R12<br>I13<br>P35<br>E78<br>S97<br>Y320	K193	G99	G11<br>R12<br>D34
D	C151<br>T183	C151<br>T152	G11<br>R12<br>I13<br>P123	K193<br>R233	T152<br>T210	P35<br>S97<br>T98<br>S121<br>P123<br>T181<br>T183

						K192

**W6V1T8 AL**	Initio		Final	
subunit	AL	NAD+	AL	NAD+
A	C151	G11<br>R12<br>I13<br>S12<br>D316	T181	F10<br>D34<br>F36
B	C151	R12<br>I13<br>D34<br>E78<br>C151<br>E317	D165<br>N166	G11<br>R12<br>I13<br>D34
C	C151<br>E317	R12<br>I13<br>S97<br>Y320	N316	R12<br>D34<br>E317
D	C151<br>H178	G11<br>R12<br>I13<br>D34	-	P190<br>K185<br>K192
