# Supplementary figures and images for: First In Silico Study of Two Echinococcus granulosus Glyceraldehyde-3-Phosphate Dehydrogenase Isoenzymes Recognized by Liver Cystic Echinococcosis Human Sera
Source: Int J Mol Sci. 2025 Oct 31;26(21):10622. doi: 10.3390/ijms262110622 (PMC12607693; doi:10.3390/ijms262110622)

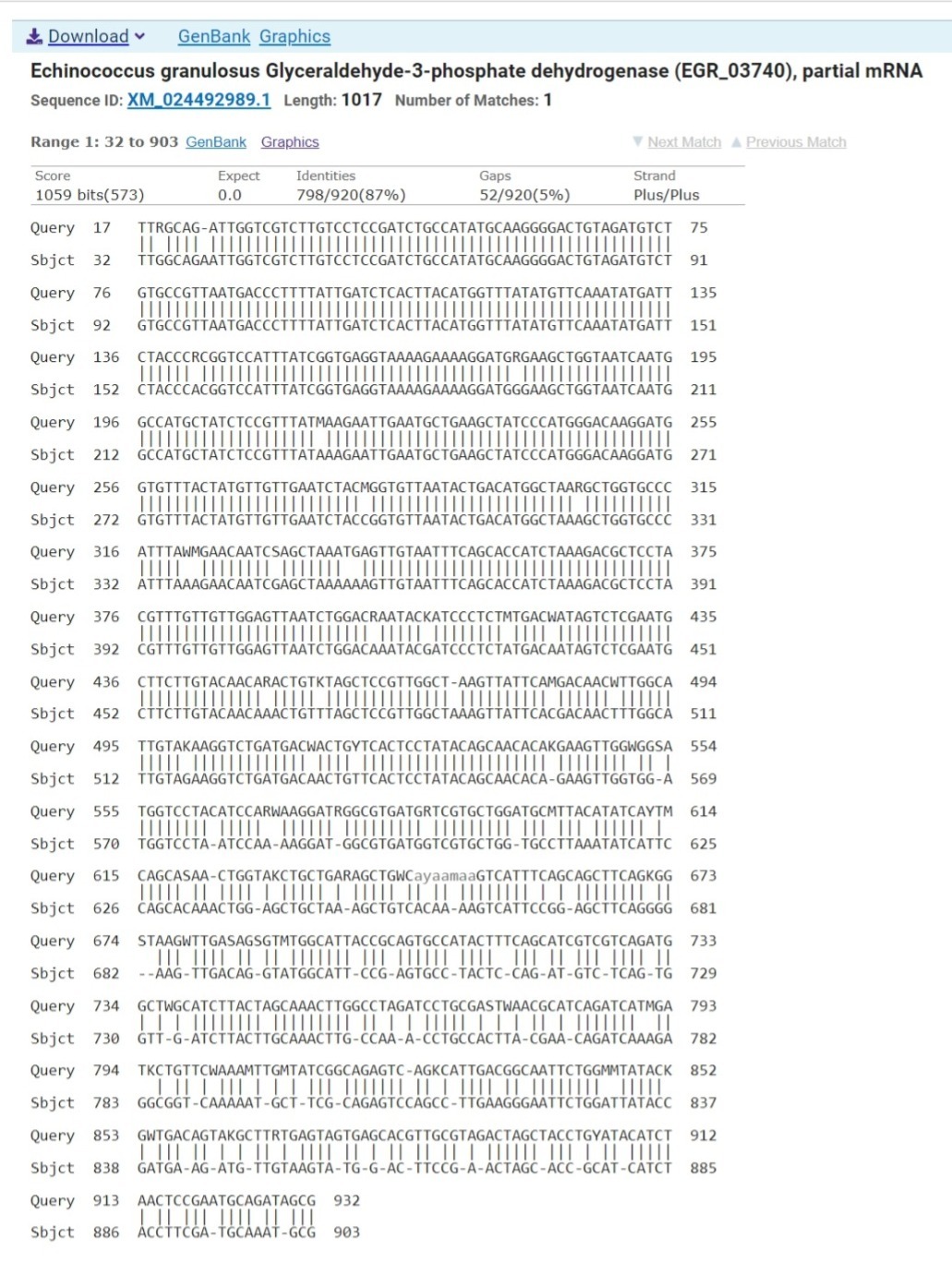

Supplement: Supplementary file 1 [file ijms-26-10622-s001.zip › FigureS1a.JPG]

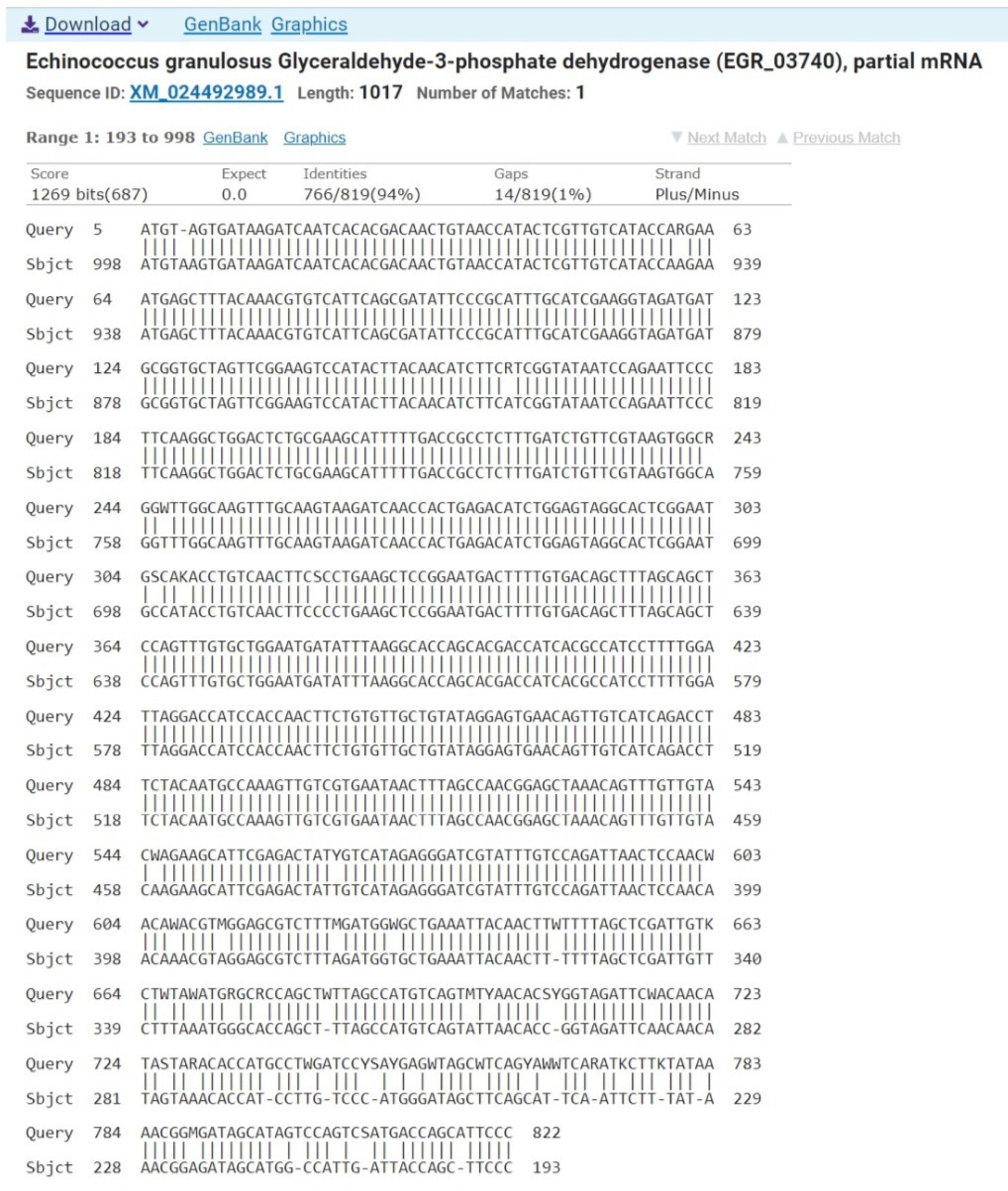

Supplement: Supplementary file 1 [file ijms-26-10622-s001.zip › FigureS1b.JPG]

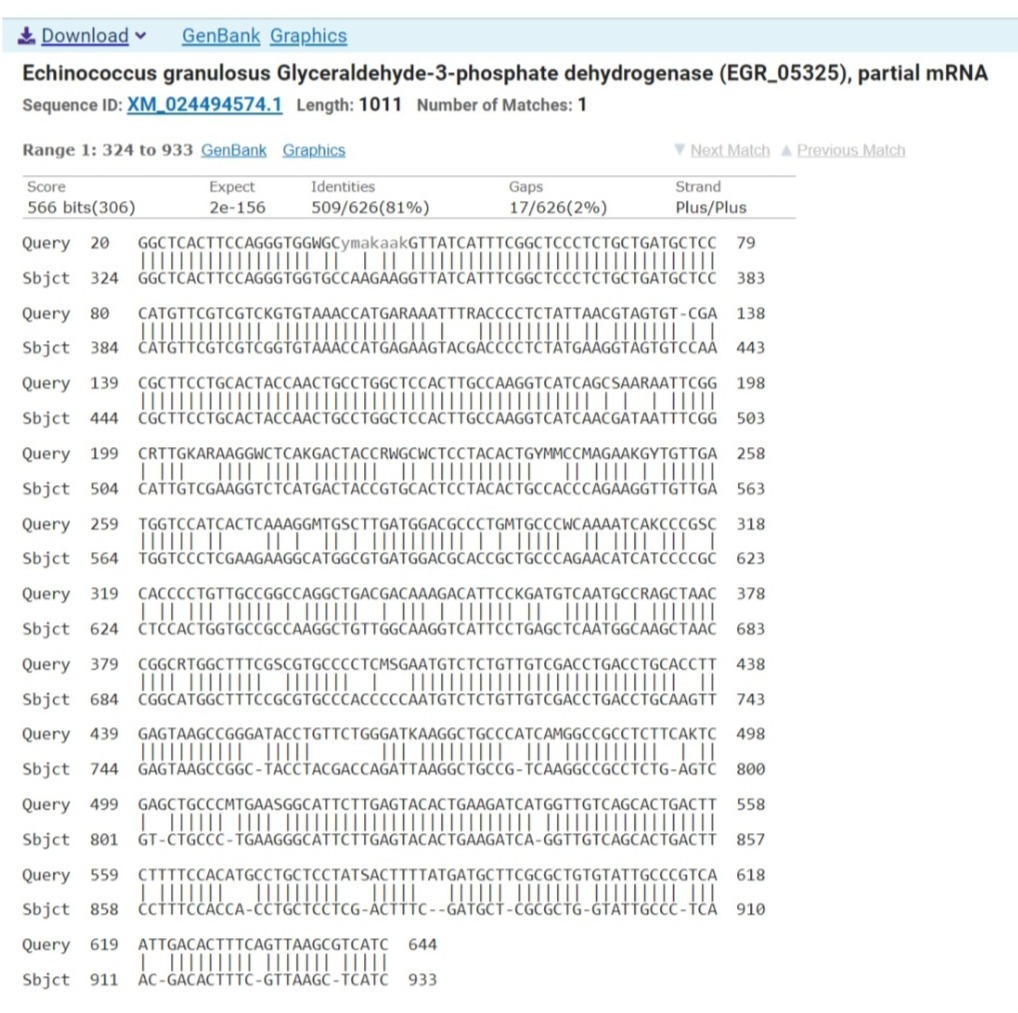

Supplement: Supplementary file 1 [file ijms-26-10622-s001.zip › FigureS1c.JPG]

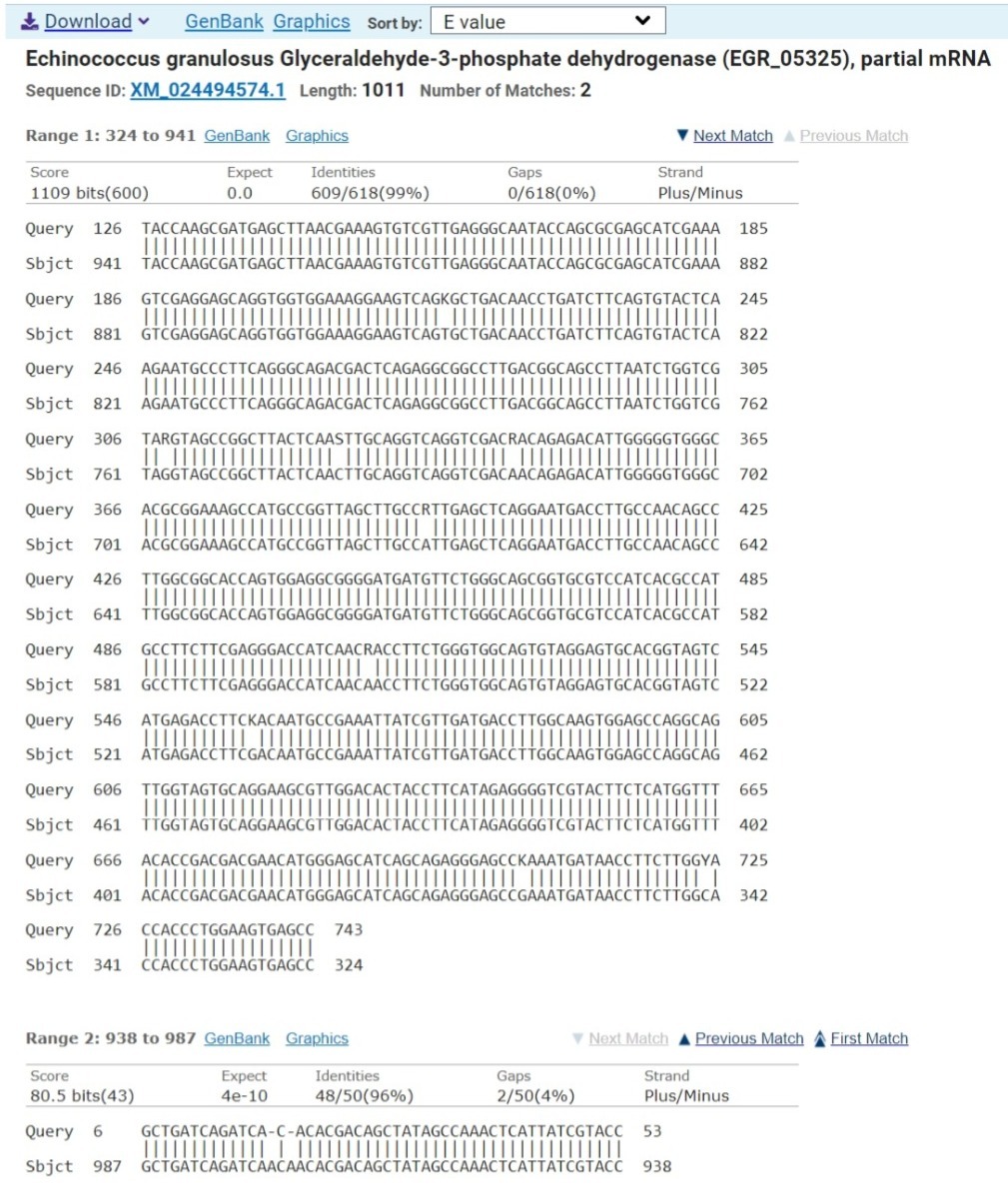

Supplement: Supplementary file 1 [file ijms-26-10622-s001.zip › FigureS1d.JPG]

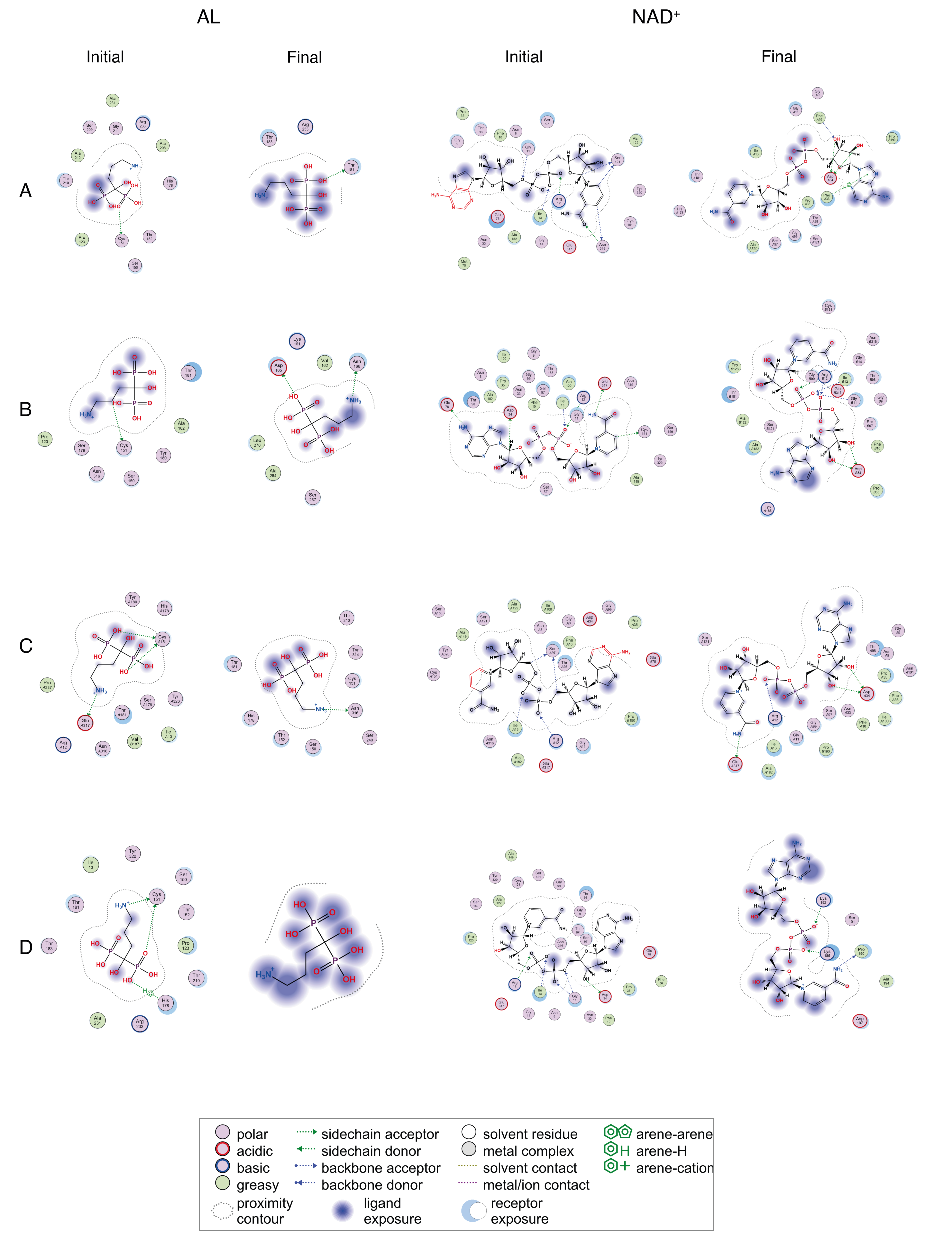

Supplement: Supplementary file 1 [file ijms-26-10622-s001.zip › S10.tif]

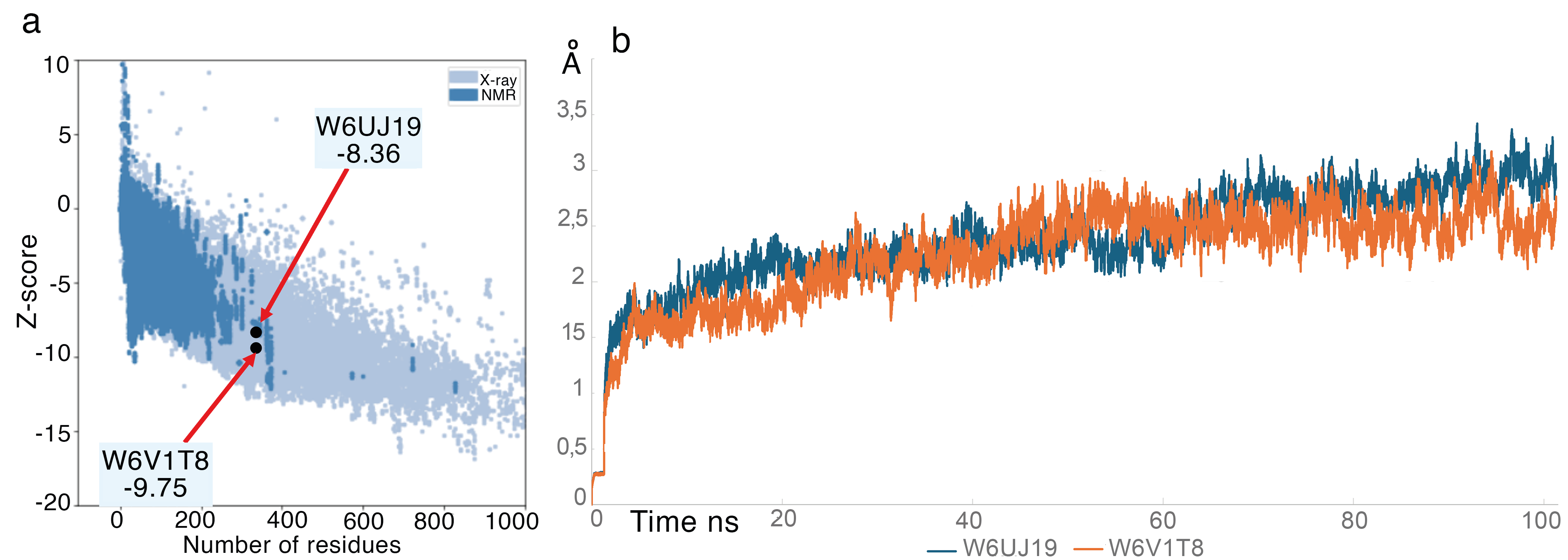

Supplement: Supplementary file 1 [file ijms-26-10622-s001.zip › S2.tif]

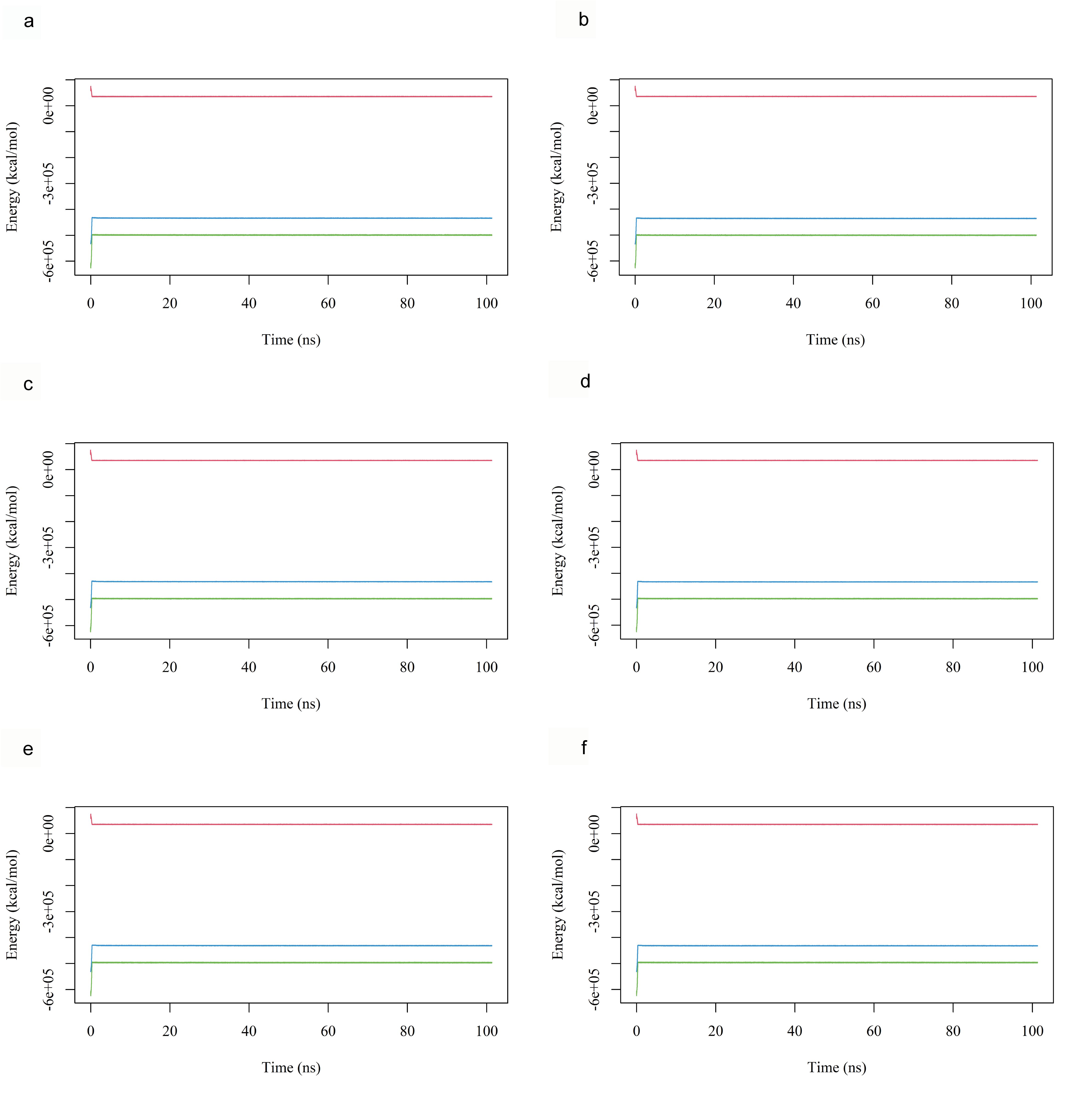

Supplement: Supplementary file 1 [file ijms-26-10622-s001.zip › S3.tif]

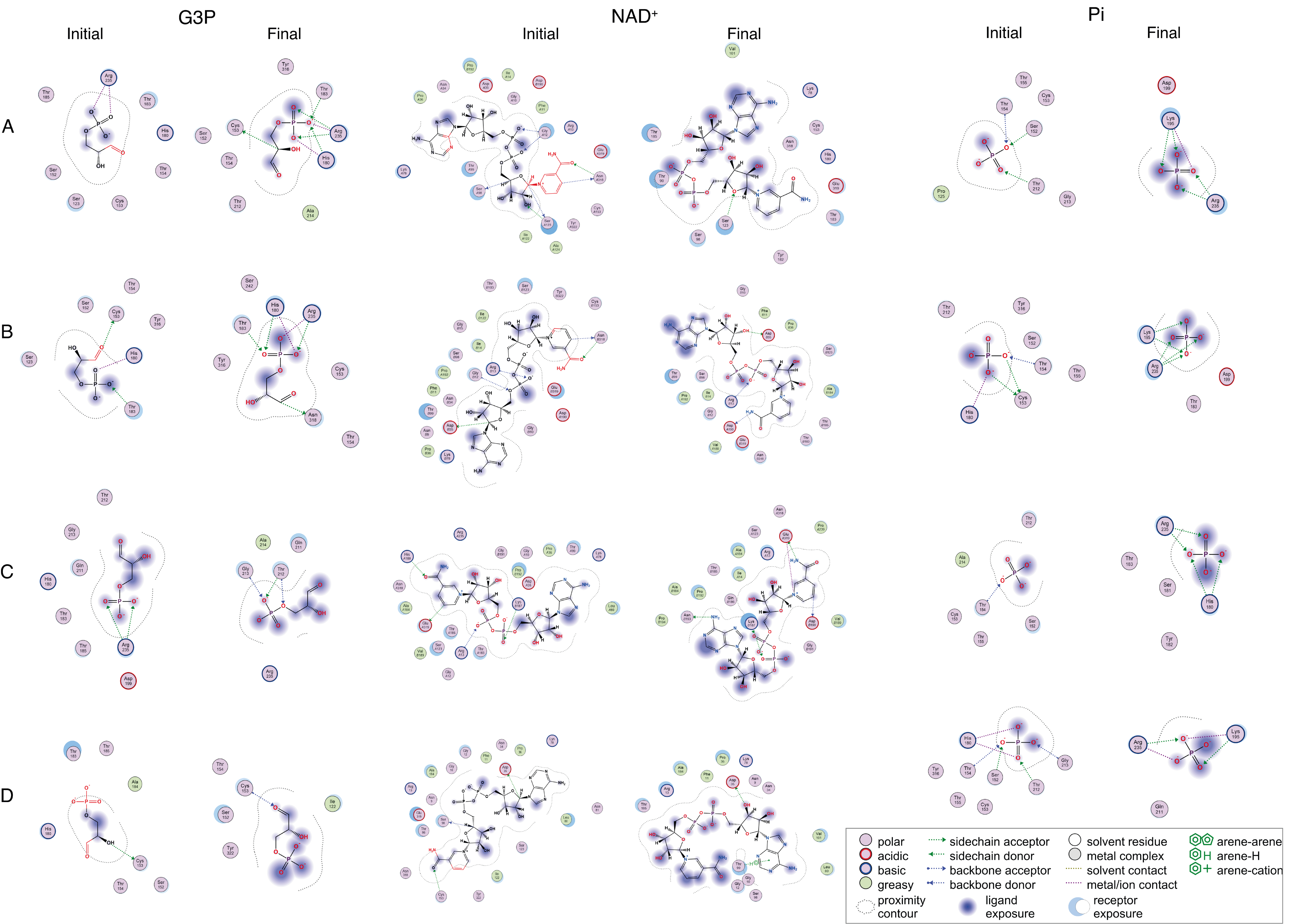

Supplement: Supplementary file 1 [file ijms-26-10622-s001.zip › S4.tif]

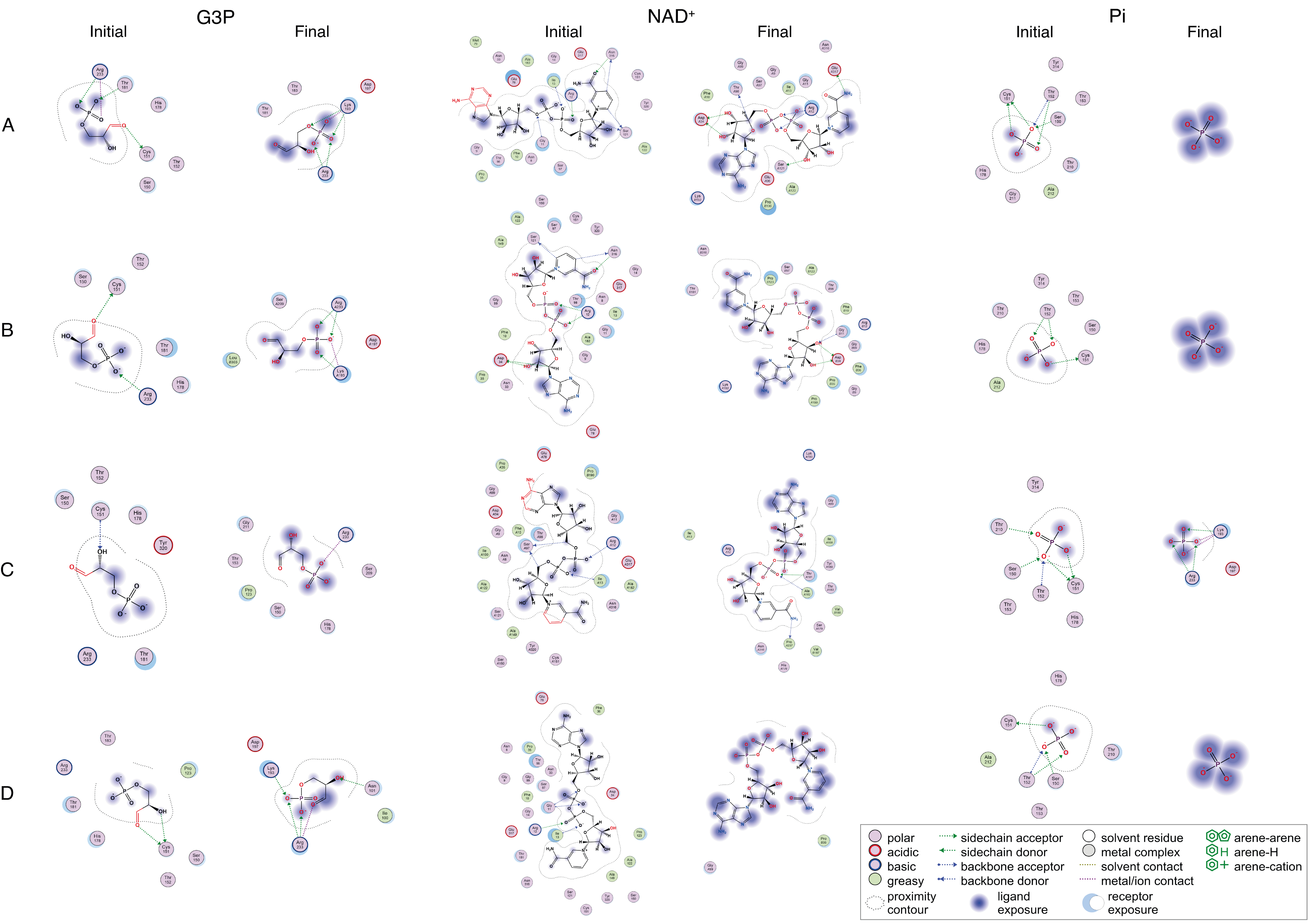

Supplement: Supplementary file 1 [file ijms-26-10622-s001.zip › S5.tif]

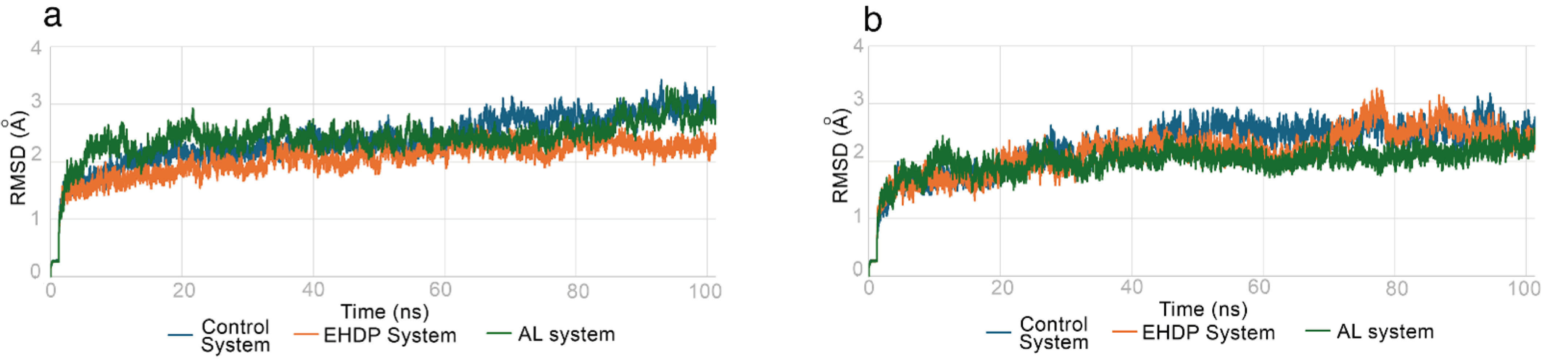

Supplement: Supplementary file 1 [file ijms-26-10622-s001.zip › S6.tif]

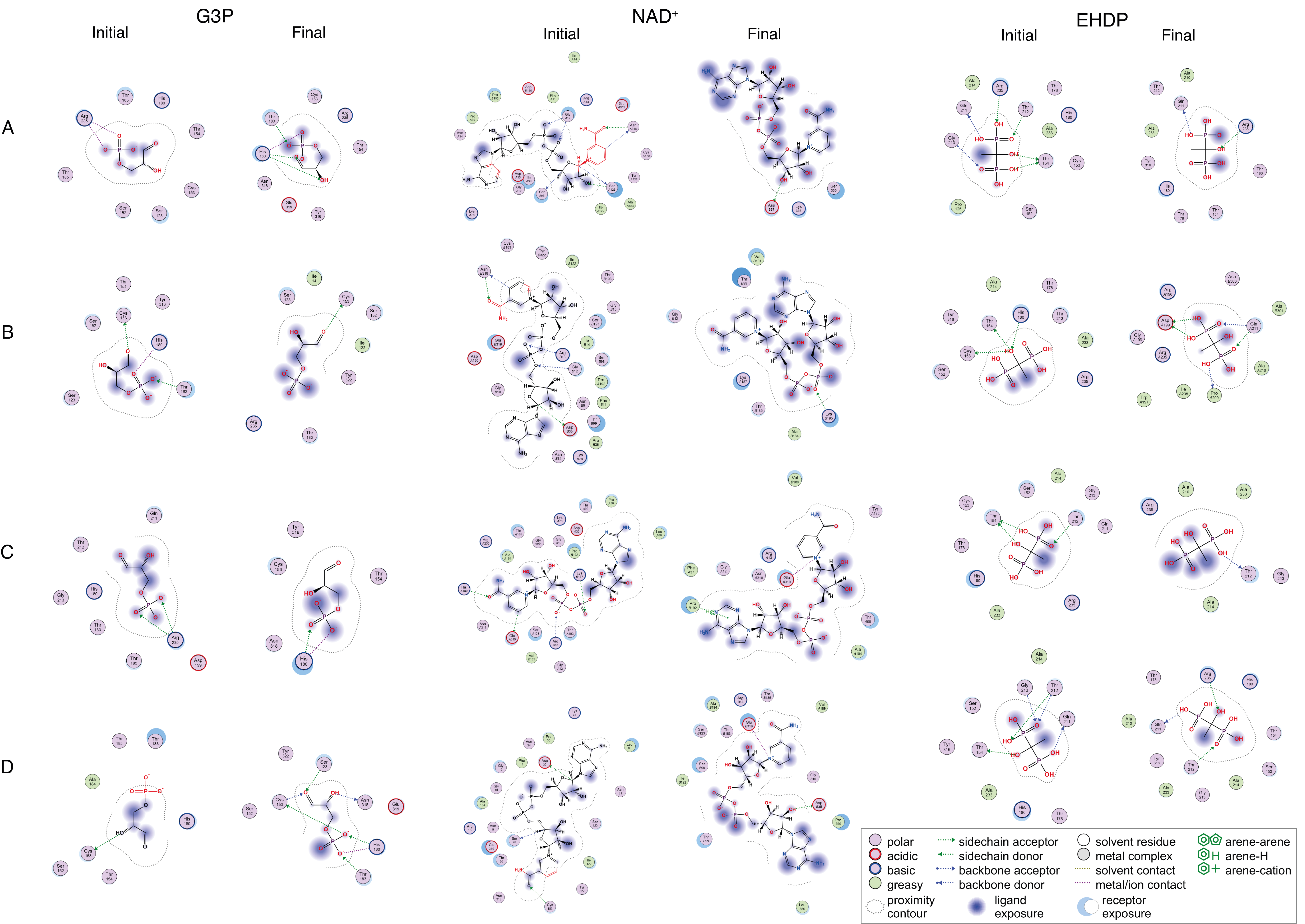

Supplement: Supplementary file 1 [file ijms-26-10622-s001.zip › S7.tif]

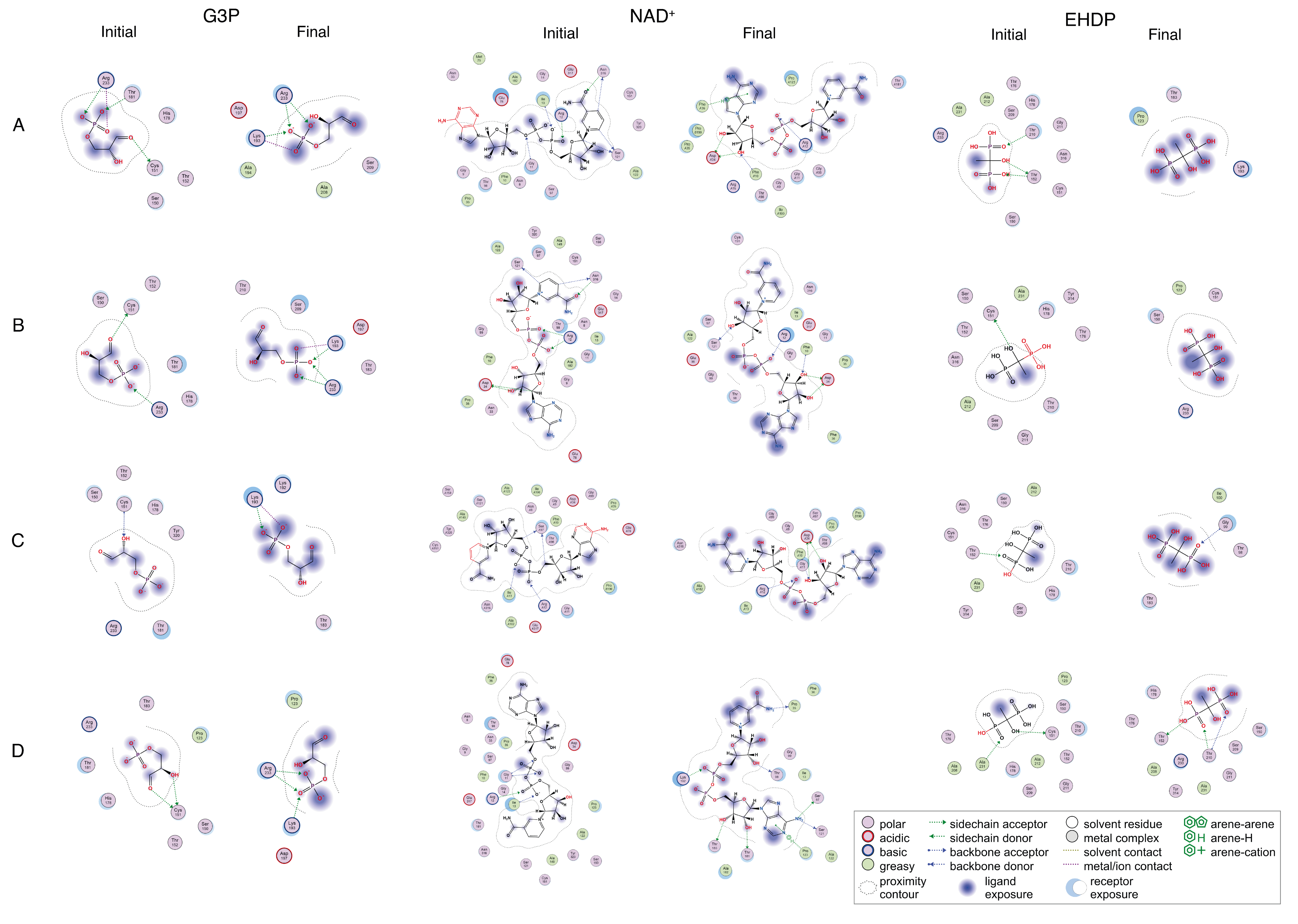

Supplement: Supplementary file 1 [file ijms-26-10622-s001.zip › S8.tif]

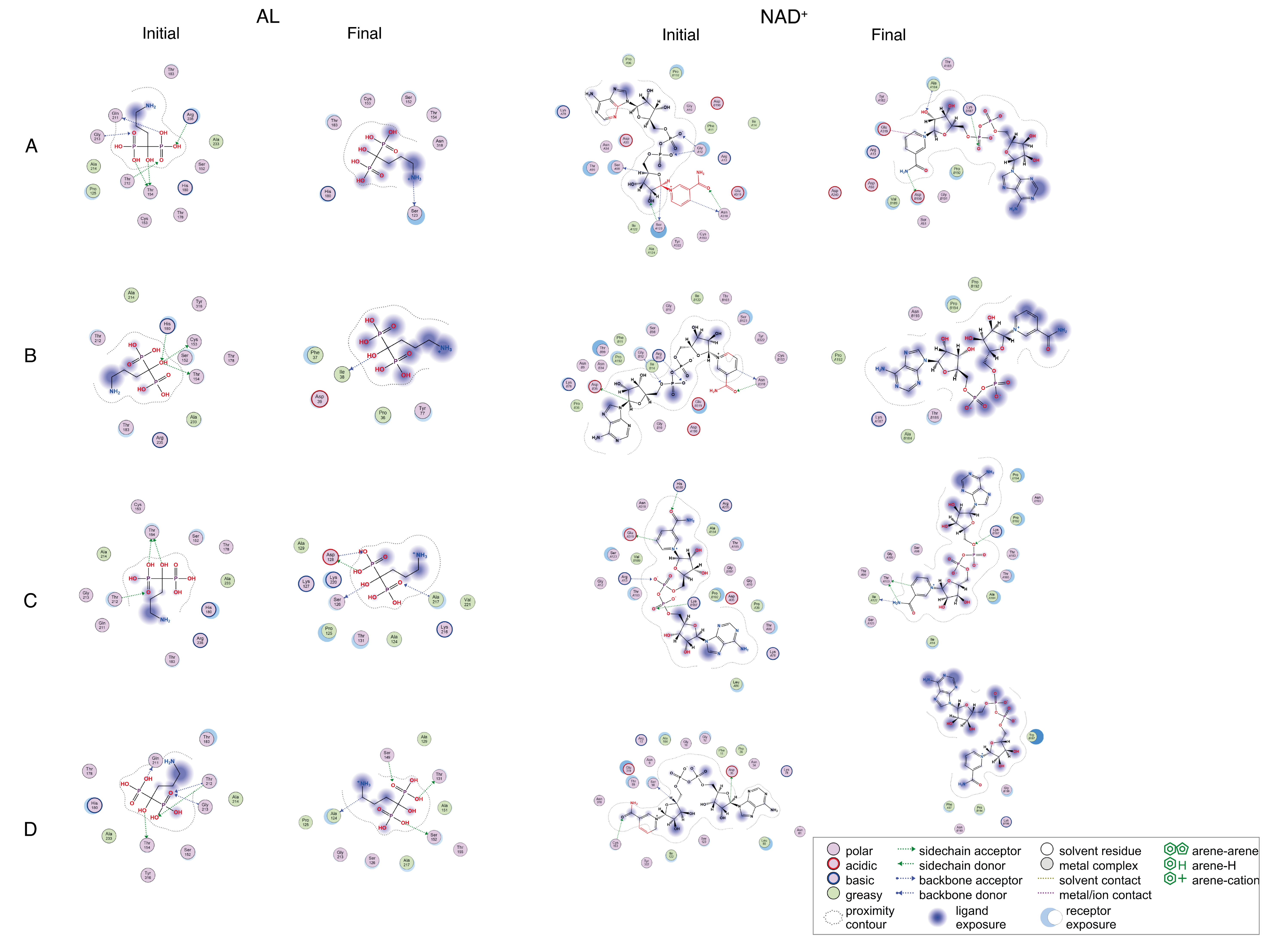

Supplement: Supplementary file 1 [file ijms-26-10622-s001.zip › S9.tif]
